# Demystifying Artificial Intelligence: A Systematic Review of Explainable Artificial Intelligence in Medical Imaging

**DOI:** 10.3390/s26072131

**Published:** 2026-03-30

**Authors:** Muhammad Fayaz, Kim Hagsong, Sufyan Danish, L. Minh Dang, Abolghasem Sadeghi-Niaraki, Hyeonjoon Moon

**Affiliations:** 1 Department of Computer Science and Engineering, Sejong University, Seoul 05006, Republic of Korea; muhammadfayaz@sju.ac.kr (M.F.); 23170856@sju.ac.kr (K.H.); sufyandanish@sju.ac.kr (S.D.); 2 The Institute of Research and Development, Duy Tan University, Da Nang 550000, Vietnam; minhdl@sejong.ac.kr; 3 Faculty of Information Technology, Duy Tan University, Da Nang 550000, Vietnam; 4 Department of Information and Communication Engineering and Convergence Engineering for Intelligent Drone, Sejong University, Seoul 05006, Republic of Korea; 5 Department of Computer Science and Engineering, XR Research Center, Sejong University, Seoul 05006, Republic of Korea; a.sadeghi@sejong.ac.kr

**Keywords:** medical image analysis, explainable AI in healthcare, explainable AI in medical imaging

## Abstract

This comprehensive literature review explores the latest advancements in explainable artificial intelligence (XAI) techniques within the field of medical imaging (MI). Over the past decade, machine learning (ML) and deep learning (DL) technologies have made significant strides in healthcare, enabling advancements in tasks such as disease diagnosis, medical image segmentation, and the detection of various medical conditions. However, despite these successes, the widespread adoption of AI-driven tools in clinical practice remains slow, primarily due to the “black-box” nature of many AI models. These models make decisions without transparent reasoning, which poses significant barriers in critical medical and legal environments, where accountability and trust are paramount. This review investigates various XAI methods, focusing on both intrinsic and post-hoc techniques, to evaluate their potential in addressing these challenges. The paper examines how XAI can enhance the transparency of healthcare algorithms, thereby fostering greater trust and confidence among clinicians, patients, and regulators. Key challenges faced by XAI in healthcare, such as limited interpretability, computational complexity, and the absence of standardized evaluation frameworks, are discussed in detail. Furthermore, this work highlights existing gaps in the literature, including the lack of detailed comparative analyses of specific XAI techniques, especially in terms of their mathematical foundations and applicability across diverse medical imaging contexts. In response to these gaps, the paper introduces a new set of standardized evaluation metrics aimed at assessing XAI performance across various medical imaging tasks, such as image segmentation, classification, and diagnosis. The review proposes actionable recommendations for enhancing the effectiveness of XAI in healthcare, with a focus on real-world clinical applications. Unlike previous studies that focus on broader overviews or limited subsets of methods, this work provides a comprehensive comparative analysis of over 18 XAI techniques, emphasizing their strengths, weaknesses, and practical implications. By offering a detailed understanding of how XAI methods can be integrated into clinical workflows, this paper aims to bridge the gap between cutting-edge AI technologies and their practical use in medical settings. Ultimately, the insights provided are valuable for researchers, clinicians, and industry professionals, encouraging the adoption and standardization of XAI practices in clinical environments, thus ensuring the successful integration of transparent, interpretable, and reliable AI systems into healthcare.

## 1. Introduction

Over the past decade, artificial intelligence (AI), powered by ML and DL, has proven highly effective in the medical field. It has been successfully applied in tasks like diagnosing breast carcinoma and brain tumors [1,2], detecting the diseases of retina [3], and performing medical image segmentation (MIS) [4,5]. Despite these successes, the widespread implementation of deep neural networks (DNN) into professional healthcare procedures has been slow. One key reason for this hesitation is the emphasis on model performance metrics rather than the explainability of the Strategic Planning process [6]. Medical professionals often hesitate to adopt AI-driven tools without understanding how the model makes decisions. Explainability is crucial in AI, particularly in healthcare, as it helps to identify model weaknesses, detect hidden patterns in data, and pinpoint irrelevant features that may affect outcomes [7]. More importantly, XAI enhances the transparency of healthcare algorithms, which fosters trust among clinicians [8]. Trust is vital, especially when AI systems inform life-or-death decisions. By making the decision-making process more understandable, XAI empowers healthcare providers to rely on AI as a supportive tool rather than a black-box system. This, in turn, boosts clinicians’ confidence in using AI for critical decisions [9,10].

XAI also plays a significant role in addressing regulatory and legal challenges in healthcare. The European Union’s General Data Protection Regulation (EU-GDPR) assigns Clarity in algorithm-driven decisions, particularly within healthcare environments [11]. The U.S. Health and Human Services Department guidelines for Clinical Decision Support Software (CDSS) similarly stress the importance of transparency and regulatory compliance to ensure AI systems are both reliable and impactful in healthcare environments. By offering explainability features, XAI helps healthcare AI systems comply with these regulatory standards, promoting both safety and efficacy while enabling informed decision-making. Furthermore, XAI mitigates potential legal risks by providing precise and understandable rationales for AI decisions, thereby supporting the accountability and auditability required in clinical care.

### 1.1. Established Works

Recognising the importance of explainability in creating authentic and trustworthy AI, scientists have conducted extensive reviews of existing XAI techniques [12]. These reviews cover a broad range of topics, including general XAI concepts, taxonomies, definitions, implementations, review of complicated models, research limitations, and standards for responsible and transparent AI, as documented in sources [13,14,15,16,17]. In research by the authors of [18,19], covering studies of 9 years (2012, 2021), across PubMed and EMBASE, the INTRPRT standard for the human-centred pattern was introduced, emphasising design fundamentals and consumer assessments. Despite these efforts, existing surveys still lack a detailed explanation of specific XAI methods in various disease contexts, as highlighted in Table 1, which summarizes the challenges faced in healthcare and the corresponding methods to overcome them [20].

**Table 1:** Comparison of Recent XAI Medical Imaging Reviews (2022–2024) and the Present Work.

Ref/Year	Focus	Comparison	Metrics	Clinical Integration	Our Work
[12] 2024	Interpretability and visualization techniques for medical imaging DL models	Reviews and categorizes XAI methods but does not compare them quantitatively	Discusses general metrics (interpretability, reliability, etc.)	Mentions clinical relevance broadly	Covers specific visualization methods; Demystifying AI provides broader taxonomy and standardized evaluation metrics
[21] 2024	Systematic review of XAI for medical image analysis	Descriptive analysis with challenges/future directions	Mentions evaluation metrics but not systematically compared	Notes clinical adoption barriers	Demystifying AI includes detailed XAI comparison and introduces new evaluation metric framework
[22] 2023	Overview of saliency-based XAI methods for clinicians	Focus on explaining saliency methods, not full comparison	Limited evaluation metrics, focused on saliency effectiveness	Oriented to clinician understanding	More focused on taxonomy and clinician lens; Demystifying AI covers multiple XAI methods across tasks
[18] 2022	Human-centered design principles for transparent ML/XAI	Reviews ML transparency, not XAI taxonomy comparison	No structured evaluation metrics	Discusses user-centric clinical design	Focused on human-centered ML; Demystifying AI focuses on XAI technique comparison and metrics specific to imaging tasks
[23] 2024	Broad XAI in healthcare (not limited to imaging)	Covers XAI generally, limited specific MI comparison	General metrics across domains	Discusses clinical applications in healthcare	Broader healthcare focus; Demystifying AI is tailored to medical imaging and specific MI tasks
**Ours**	**Systematic review of XAI in medical imaging (segmentation, classification, diagnosis)**	**Detailed comparative analysis of 18+ XAI techniques**	**Standardized evaluation metrics tailored to MI XAI**	**Emphasizes practical integration and clinical workflow**	**Provides deeper method comparison, mathematically grounded analysis, and novel evaluation metrics across imaging contexts**

In addition, research by Patricio and Messina et al. [24,25] has examined the latest progress in explainable deep learning (DL) for medical imaging, including both post-hoc and intrinsic methods such as concept bottleneck models, ProtoPNet, and attention mechanisms. A comparison between intrinsic and post-hoc interpretations for convolutional neural networks (CNNs) was presented in [26], which also featured a taxonomy of XAI and outlined directions for future research.

However, these reviews do not sufficiently explore the strengths and weaknesses of different XAI techniques, especially in terms of their mathematical underpinnings. While we acknowledge that some prior works, such as those by Patricio et al. and Messina et al. [24,25], provide a broader range of techniques including intrinsic models, our focus remains on analyzing their comparative depth in medical imaging tasks. Researchers in [27] provided a systematic review of XAI’s role in addressing the pandemic, exploring its uses in data enhancement, result prediction, unsupervised clustering, and image partitioning. Despite this, they do not critically examine the performance of specific XAI methods in different medical imaging scenarios. Furthermore, the authors in [28,29] explored the use of XAI for classifying DL-based image analysis methods and conducted a survey of XAI papers up until the end of 2024. However, they did not explore the technical details or mathematical basis of the methods they reviewed, instead focusing on a limited number of techniques. It should be noted that we critique the lack of performance analysis in some prior works. Our review also primarily emphasizes methodological descriptions over quantitative performance comparisons, which is a limitation we aim to address in future work. These surveys also overlook the practical challenges of implementing XAI in clinical practice, such as interpretability across diverse patient populations.

Other reviews, such as those by [22], categorized XAI methods into saliency-based approaches, with [30] expanding the discussion to include non-saliency-based methodologies. For example, the work by Messina et al. [25] is more narrowly scoped on explaining medical report generation, which differs from our focus on general medical imaging tasks. While this research has explored foundational XAI concepts, classifications, and the use of interpretable DL in MI, especially post-hoc methods, it does not provide comprehensive evaluations of how these methods perform in real-world medical settings or specific guidelines for evaluating different XAI techniques in diverse imaging contexts. This paper addresses these gaps by providing a detailed comparative analysis of various XAI approaches, with a focus on their mathematical foundations, strengths, weaknesses, and practical considerations in the context of medical imaging. We synthesize existing evaluation approaches and propose practical guidance for selecting appropriate metrics based on clinical context, imaging modality, and diagnostic task requirements. Rather than proposing a novel framework, this review provides a comprehensive analysis of evaluation methodologies employed in recent XAI studies, identifying strengths, limitations, and key trends to inform future clinical validation efforts.

1. Lack of Detailed Discussion on Specific XAI Methods: Previous reviews, such as the one in [18], focus on general design principles but do not delve into the specifics of different XAI methods or their applicability in various disease contexts or data types. This paper provides a detailed analysis of specific XAI methods, emphasizing their strengths and weaknesses in the context of medical imaging, particularly in different disease types and data modalities.
2. Limited Examination of Mathematical Underpinnings and Practical Challenges: Several studies, including [24,25], review XAI techniques but fail to provide a detailed investigation into their mathematical foundations and the challenges of applying them in real-world clinical scenarios. This paper presents a comparative analysis of XAI techniques, focusing on their mathematical foundations and providing practical recommendations for addressing challenges in their real-world applications.
3. Insufficient Evaluation of XAI’s Performance Across Medical Imaging Scenarios: Existing reviews often overlook the effectiveness of XAI methods in diverse medical imaging scenarios, including variations in patient demographics and image types. This paper presents a systematic review of existing XAI methods applied to medical imaging across various disease contexts. This review provides a comprehensive analysis of evaluation approaches used in XAI methods within medical imaging over the past few years, identifying strengths, limitations, and key trends, rather than proposing a novel framework.

To further clarify our contributions, we present a gap analysis Table 2 that contrasts the limitations of previous studies with the gaps addressed in our work. This comparison highlights our focus on providing a taxonomy of XAI methods, emphasizing mathematical underpinnings and discussing practical implications across various medical imaging scenarios.

### 1.2. Aim of the Review

This study differs from existing literature by aiming to address an important research gap through a comprehensive review of XAI techniques specifically applied to medical imaging. Unlike previous studies that may have focused on narrower aspects of the field, this research integrates a wide range of evaluation metrics, relevant diseases, and suitable datasets, providing a more holistic understanding of how XAI can be utilized in medical imaging. The review also examines the strengths and weaknesses of each XAI method, as well as the challenges they present. By critically examining these factors, the study offers recommendations for improving or refining the techniques.

Moreover, the paper provides a comparative analysis of various XAI approaches, exploring both their mathematical foundations and operational procedures. This comparative evaluation enables readers to understand the differences and similarities between the methods, thereby guiding the selection of the most suitable approach for various medical imaging tasks. In addition to the current state of XAI in medical imaging, the paper highlights promising directions for future research, aiming to advance the field and improve its application in healthcare. By doing so, it seeks to provide valuable insights not only to researchers and developers but also to clinicians, medical professionals, and patients, all of whom can benefit from more transparent and interpretable AI models in medical imaging.

## 2. Related Study

The Related Study section discusses an in-depth history of applying XAI in MI. It also defines various types of medical imaging (MI), including MRI, endoscopy, X-rays, CT scans, and fundus images. The development of explainable expert systems dates back to the mid-1980s [31], although the term “XAI” was first coined by [32] in 2004. The significance of XAI has grown dramatically with the rise of deep learning models used in the industry. The Advanced Research Projects Agency [9] released the XAI initiative to promote the creation of different systems that are not just interpretable but also foster greater trust and confidence within the community through improved transparency and interpretability [31]. Around the same time, the European Union passed regulations regarding the “right to algorithmic explanations,” ensuring that everyone has the right to understand the Evaluation process behind algorithms that use their data [33]. This legal shift led to a renewed focus in research on establishing models that prioritize explainability as much as, or more than, accuracy. Consequently, interest in XAI has surged within the scientific community, with a significant increase in relevant scholarly works in recent years [34]. To fully grasp XAI, it is necessary to acknowledge some foundational concepts:

- **Explainability:** Explainability refers to the capability of an AI system to make its decision-making process transparent, understandable, and traceable to humans. It goes beyond simply presenting the model’s output; it aims to provide clear and structured reasoning that explains why a particular prediction or action was made. This involves revealing the internal logic, feature contributions, or decision pathways used by the model, enabling users to understand how input data is transformed into an output. By making the inner workings of the AI system accessible, explainability allows clinicians, researchers, and developers to examine the rationale behind model decisions. Explainability is therefore considered a property of the model or the XAI method itself, as it focuses on generating faithful and transparent representations of the model’s behavior. This clarity is essential for validating model reliability, identifying biases, and ensuring that predictions are based on meaningful and clinically relevant patterns rather than spurious correlations. In medical imaging and healthcare applications, explainability plays a critical role by providing evidence for AI-driven decisions, such as highlighting regions of interest (e.g., tumors or lesions) that influence a diagnosis. Ultimately, explainability supports transparency, accountability, and trust by enabling users to inspect and verify model reasoning, making it a fundamental requirement for the safe deployment of AI systems in high-stakes domains such as healthcare.
- **Interpretation:** Interpretation refers to the human-centered process of understanding, analyzing, and assigning meaning to the explanations and outputs generated by an AI system. Unlike explainability, which focuses on producing explanations, interpretation depends on how users perceive, evaluate, and utilize those explanations within a specific context. It involves translating model outputs or explanatory representations into actionable insights that are meaningful to the user. This process helps bridge the gap between complex computational models and human reasoning, enabling users to comprehend why a model produces certain outcomes. Interpretation is often facilitated through tools such as visualizations (e.g., heatmaps), feature importance rankings, or natural language descriptions, which help make model behavior more accessible. However, interpretation is inherently subjective and may vary depending on the user’s expertise, domain knowledge, and application context. In medical applications, interpretation allows clinicians to relate model explanations to clinical knowledge, such as understanding how highlighted image regions correspond to pathological features. This enables practitioners to validate AI predictions and integrate them into decision-making processes. Thus, interpretation transforms explainable outputs into meaningful, context-aware understanding that supports practical use and informed decision-making.

To further clarify the distinction, explainability refers to the capability of an AI system to generate transparent and understandable reasoning for its predictions, whereas interpretation refers to how a human user understands and assigns meaning to those explanations. In this sense, explainability is a property of the model or method, while interpretation depends on the user’s perspective, domain expertise, and application context. For example, a saliency map generated by an XAI method provides explainability, whereas a clinician analyzing that map to understand the presence of a tumor represents interpretation.

- **Reliability:** Reliability in XAI denotes the reliability and stability of the explanations and predictions generated by an AI model. A reliable XAI system ensures that the model produces consistent outcomes when presented with similar inputs or conditions, meaning that users can count on it to give stable results. This consistency is essential for fostering confidence in the model’s behaviour and ensuring that its decisions are not random or subject to fluctuations. A reliable system reassures users that its explanations can be trusted, as they will remain predictable and coherent over time. For an XAI model to be truly reliable, it must offer repeatable results and maintain the same level of accuracy and clarity regardless of when it is used. This consistency is vital for applications where decisions based on AI predictions have real-world consequences, such as in healthcare or finance. If a model’s explanations shift unpredictably or lead to different conclusions under similar conditions, it can undermine user trust and the effectiveness of the system. Therefore, reliability ensures that explanations provided by XAI are not only accurate but also stable and dependable, creating a solid foundation for ongoing use and trust in the technology.
- **Robustness:** Robustness refers to the ability of an AI model to maintain its accuracy and deliver reliable explanations, even when faced with disruptions such as noise, data changes, or adversarial attempts. A robust XAI system is designed to remain effective and consistent, even if the input data is altered or corrupted. This means that, regardless of unexpected variations or attempts to deceive the system, the model should continue to perform accurately and offer trustworthy explanations. The resilience of the system ensures that users can rely on it to make decisions and interpret its reasoning, regardless of external challenges. For an XAI system to be genuinely robust, it must handle a wide range of possible scenarios and data fluctuations without losing its ability to provide clear, meaningful insights. Whether the model is exposed to minor errors, slight changes in input, or even deliberate attempts to manipulate its output, it should still deliver precise and relevant explanations. This resilience is critical in sensitive fields like healthcare [35] or security, where even minor disruptions in the data could have significant consequences. Robustness ensures that the AI model remains stable, trustworthy, and valuable in real-world applications, offering explanations that remain valid even in challenging conditions.

### 2.1. Taxonomy of XAI Methods in MI

**Figure 1:**
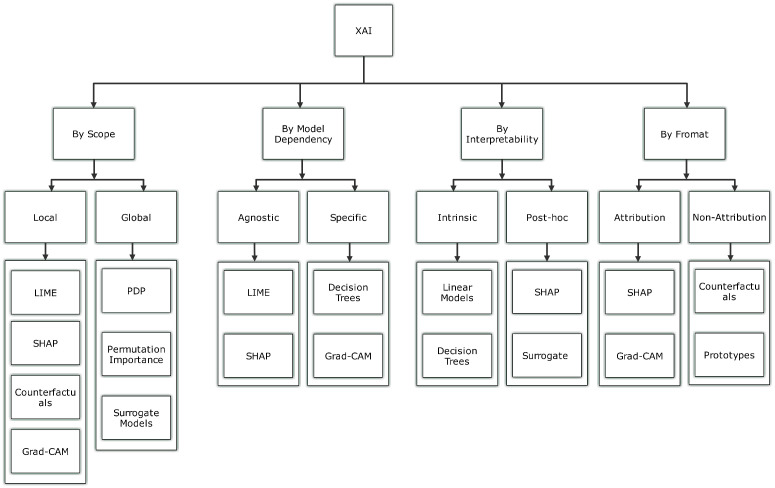
Adopted framework for categorizing XAI methods.

Explainability in Artificial Intelligence (AI) is pivotal for understanding how machine learning (ML) and deep learning (DL) models make decisions. In the context of medical imaging (MI), explainability is essential to ensure that AI models are transparent, interpretable, and trusted by clinicians. To better understand the diversity of XAI techniques in MI, we categorize these methods into several distinct classes, as shown in the updated taxonomy in Figure 1.

In this review, we provide an updated taxonomy that includes both traditional approaches and emerging methods, addressing various complexities in the state-of-the-art of XAI. These categories help to clearly classify methods based on their operational characteristics.

**Figure 2:**
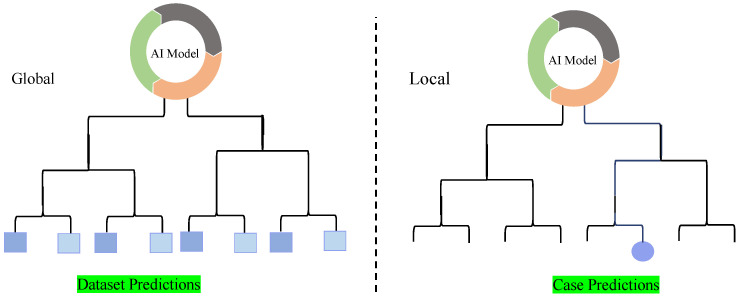
Schematic representation of the AI model and its components. The AI model is depicted as a colored circle box, representing its core structure. Arrows indicate the flow of data between different components, and the boxes represent key stages or features within the model. All graphical elements are consistently designed with solid-colored boxes to enhance clarity and visual cohesion.

**Figure 3:**
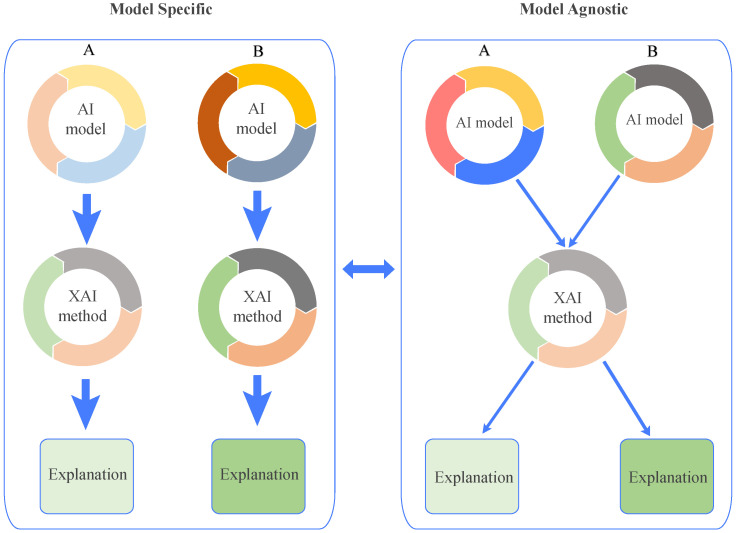
Schematic representation of Explainable AI (XAI) methods categorized by their dependencies: model-specific methods tailored to particular machine learning models and model-agnostic methods applicable across diverse model types.

- **Attribution and Non-Attribution Explanations:** Attribution and non-attribution explanations are two distinct approaches used in XAI to shed light on the decision-making process of ML and DL models [36]. Attribution methods focus on identifying and visualising specific parts of the input data, such as areas of an image, that significantly influence the model’s prediction. These methods often use techniques like localization maps to visually highlight which regions of the image are most responsible for the model’s output. While these methods are valuable for locating areas of interest, they do not provide detailed insights into which specific features (e.g., color, texture, or shape) within those regions drive the decision. Therefore, attribution offers a partial rather than a complete understanding of the model’s reasoning. By drawing attention to these key areas, attribution provides a more direct understanding of how the model reaches its conclusions. In contrast, non-attribution methods take a broader approach by seeking to uncover the overall reasoning behind the model’s decisions. Rather than focusing on individual data points, such as pixels in an image, these methods explore the larger dynamics at play, such as the model’s sensitivity and stability in different conditions. Non-attribution techniques are beneficial for understanding the general behavior of the model and diagnosing potential issues. They offer valuable insights that can aid in model debugging, refinement, and ensuring that the system functions reliably across various scenarios [37]. By examining the model’s underlying processes, non-attribution methods contribute to improving the model’s performance and interpretability more holistically.
- **Local and Global Explanations:** Local and global explanations are crucial in shedding light on how ML and DL models make decisions, bridging the gap between human understanding and machine reasoning [38]. The local explanation approach focuses on interpreting the logic behind the model’s selections for specific data instances, revealing how specific input elements impact the decision positively or negatively. In contrast, global explanations aim to provide a comprehensive understanding of the model’s overall behaviour, offering a broader view of its logic. For instance, pinpointing the crucial factors that influence the model’s overall performance is a key aspect of the global explanation approach. Figure 2 provides a schematic representation of local and global explainability methods, showcasing their unique perspectives in interpreting machine learning models at both granular and overall levels [39].
- **Intrinsic and Post-Hoc Explanations:** Intrinsic and post-hoc explanations are fundamental approaches [40] used to clarify the internal mechanisms of ML and DL models. Intrinsic approaches are embedded within the structure of the model itself, providing built-in interpretability. These methods are typically used with models like decision trees and rule-based systems [32,41], where the decision-making process is inherently easier to understand due to the model’s structure. By their nature, these techniques make the model’s logic more transparent, allowing users to see how inputs lead to specific outputs. On the other hand, post-hoc explanations are applied upon completion of model training and are external to the model’s structure. These techniques don’t alter the original model but instead provide insights into its behaviour and predictions. Post-hoc methods can be used with a variety of pre-trained models, such as CNNs and Vision Transformers (ViTs), without affecting their performance or accuracy [42]. These approaches offer flexibility, enabling interpretability even for complex models that are not inherently interpretable, making them valuable tools for understanding and trusting AI decisions.
- **Model-Specific and Model-Agnostic Explanations:** In XAI, building credibility and maintaining transparency requires a clear understanding of how ML and DL models make decisions, which is achieved through model-specific and agnostic approaches [43]. The model-specific approach is tailored to the unique architecture and parameters of a particular model, providing explanations based on its specific structure. On the other hand, the agnostic approach is independent of the model’s architecture, making it versatile and applicable to any other different domains without needing to interact with the model’s parameters directly [44]. Figure 3 presents a schematic representation of model-agnostic and model-specific explainability methods, highlighting their distinct approaches to interpreting and understanding machine learning models.
- **Case-Based Explanations:** Case-based explanations involve comparing new data to similar past cases to provide clarity on how past examples influenced the current decision. This approach helps in identifying patterns or similarities between the current instance and previous ones, making the decision-making process more transparent. By leveraging the outcomes of past cases, these methods can explain why a particular decision was made based on the resemblance to previous instances. An example of this technique is ProtoPNet (Prototypical Part Networks), which identifies and compares key parts of an image (or data) to prototypical examples that are known to influence a model’s decision. This comparison helps clarify the reasoning behind a prediction by showing how features from earlier cases are used to inform the current decision.
- **Concept-Based Explanations:** Concept-based explanations focus on providing a higher-level understanding of a model’s decision-making process by associating predictions with underlying, often abstract concepts such as learned features (i.e., intermediate feature-map activations in deep neural networks capturing patterns such as edges, textures, or anatomical structures) or predefined medical concepts (e.g., tumors, lesions). This approach helps to interpret a model’s predictions by linking them to the broader medical or domain-specific concepts that the model has learned during training. Concept Activation Vectors (CAVs) are a popular example of this method, as they allow for a clearer understanding of how the model interprets certain medical conditions or features (like detecting a tumor) based on high-level concepts. By leveraging these concepts, medical professionals can gain insight into how a model links data to specific, meaningful medical features, helping to improve the model’s interpretability and trustworthiness.
- **Counterfactual Explanations:** Counterfactual explanations are designed to identify the minimal changes required to alter a model’s decision, providing valuable insights into the model’s decision-making process. By exploring “what-if” scenarios, counterfactuals examine what would happen if specific input features were altered. These explanations help highlight which features (e.g., pixel regions, feature-map activations, or clinically derived attributes depending on the method) had the most significant impact on the model’s predictions. For example, a counterfactual explanation might reveal that changing a particular medical image feature (such as the size of a tumor) could shift the model’s classification of the disease. This method allows stakeholders, especially clinicians, to better understand model decisions and consider the possible changes that could influence an outcome. Counterfactuals provide actionable insights by offering explanations of how small modifications to input data can lead to different predictions, thereby helping to improve both model accuracy and user trust.
- **Natural Language Explanations:** Natural language explanations enhance the interpretability of AI models by converting complex, often technical model outputs into human-readable text. These explanations describe the model’s decision-making process in terms that are easily understood by non-experts, including clinicians who may not have deep technical expertise in AI. In medical imaging, natural language explanations are especially valuable, as they allow clinicians to better understand the reasoning behind an AI-driven diagnosis or recommendation. For example, after analyzing an MRI scan, an AI model might generate a textual explanation that outlines the key features (e.g., tumor size and location, typically derived from segmented regions rather than individual pixels) that led to a particular diagnosis. This approach makes AI tools more accessible and fosters trust among healthcare providers by ensuring that the decision-making process is transparent and understandable, helping to integrate AI more effectively into clinical practice.

### 2.2. Terminology Clarification: Pixels vs. Features in XAI

To avoid ambiguity in subsequent sections, we clarify our usage of key terms:

- **Pixel**: Refers to the atomic unit of a digital image (e.g., a single (x,y) location in a 2D radiograph or (x,y,z) voxel in a 3D CT volume). In gradient-based XAI methods (e.g., saliency maps), attribution scores are computed per pixel to indicate local influence on model output.
- **Feature**: Denotes higher-level representations derived from groups of pixels through convolutional operations. In CNNs, features correspond to activation values in intermediate layers that encode semantic concepts (e.g., edges, textures, anatomical structures). Methods like Grad-CAM compute attributions at the feature map level before upsampling to pixel space.
- **Clinical implication**: Pixel-level explanations provide fine-grained localization but may lack semantic meaning (e.g., highlighting individual pixels within a tumor boundary). Feature-level explanations offer more clinically interpretable rationales (e.g., “the model focused on spiculated margins”) but sacrifice spatial precision. Optimal XAI deployment requires matching explanation granularity to clinical task requirements.

In this work, the term “feature” is used at multiple abstraction levels depending on the XAI method. At the lowest level, features correspond to individual pixels or voxels. At an intermediate level, features refer to learned representations within convolutional layers (feature maps), which capture patterns such as edges, textures, or anatomical structures. At a higher level, features may represent semantically meaningful or clinically derived attributes (e.g., tumor size, location, or shape), which are often computed from segmented regions rather than raw pixels. To avoid ambiguity, we explicitly specify the feature abstraction level when describing each XAI method in the following sections.

### 2.3. XAI Techniques in Medical Data

XAI methods are playing a significant role in medical imaging by making the decision-making processes of ML and DL models more understandable and transparent [45]. These approaches help bridge the difference between human intuition and the advanced logic used by models, especially when dealing with visual data like MI. XAI techniques pinpoint key areas within images that the model focuses on, giving medical professionals a more precise understanding of how the model derives its decisions. This makes the otherwise complex decision-making process easier to grasp. As discussed in the Introduction and outlined in Table 1, the challenges to the adoption of XAI in healthcare, such as regulatory compliance, trust, and transparency, are addressed through various methods, contributing to the effectiveness of XAI in medical imaging [21].

**Figure 4:**
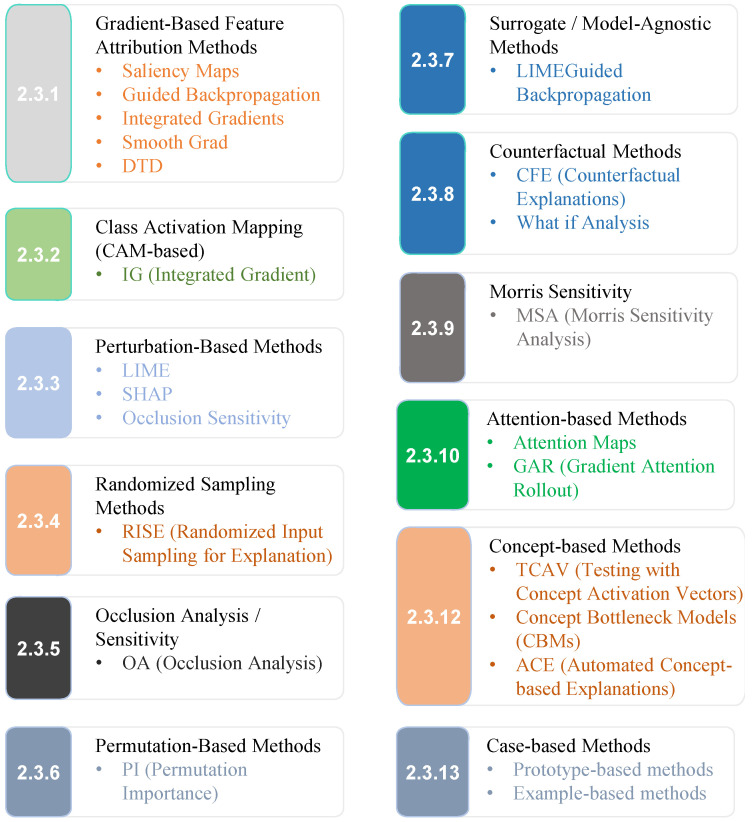
XAI methods based on medical data.

**XAI-Based Methods Applied to Medical Data**: Enhancing Interpretability and Transparency in Diagnoses Across Modalities is illustrated in Figure 4. Additionally, counterfactual methods, which present similar images that generate distinct outcomes from the DL models, enhance interpretability by offering a contrasting perspective. These techniques are increasingly used in MI analysis, and various approaches from recent studies have been applied and discussed in detail to improve the transparency of AI-driven decisions in the field [46].

#### 2.3.1. Gradient-Based Feature Attribution Methods

Gradient-based methods work by calculating the importance of each input feature, typically at the pixel or voxel level, by analyzing the gradient of the model output with respect to the input image. Essentially, these methods highlight the areas or features of the input data that most influence the model’s prediction. These techniques are essential for creating visual saliency maps that help to understand which parts of an image are critical for decision-making. By backpropagating gradients through the model, they identify the most influential features in the decision-making process. Saliency Maps visualize the gradient of the model’s output with respect to the input image, highlighting areas that have the greatest influence on the model’s prediction. These maps identify which regions in the image the model is focusing on to make a decision, offering a clear representation of important features contributing to the final output. Integrated Gradients are an enhanced version of saliency maps that integrates gradients over multiple input values, providing a more accurate and stable attribution of importance to different features. By reducing noise and accumulating gradient information, it helps to highlight the most critical features in a more interpretable and robust manner, making it especially useful in complex models. Grad-CAM (Gradient-weighted Class Activation Mapping) generates heatmaps that visually highlight the regions of an image most significant for the model’s predictions. By using the gradients of the target class with respect to the feature maps of a convolutional layer, Grad-CAM provides a clear localization map, showing which areas of the image are influencing the model’s classification decision, enhancing interpretability and trust. These methods primarily operate at the pixel-level, although the gradients may be computed through intermediate feature maps.

**Figure 5:**
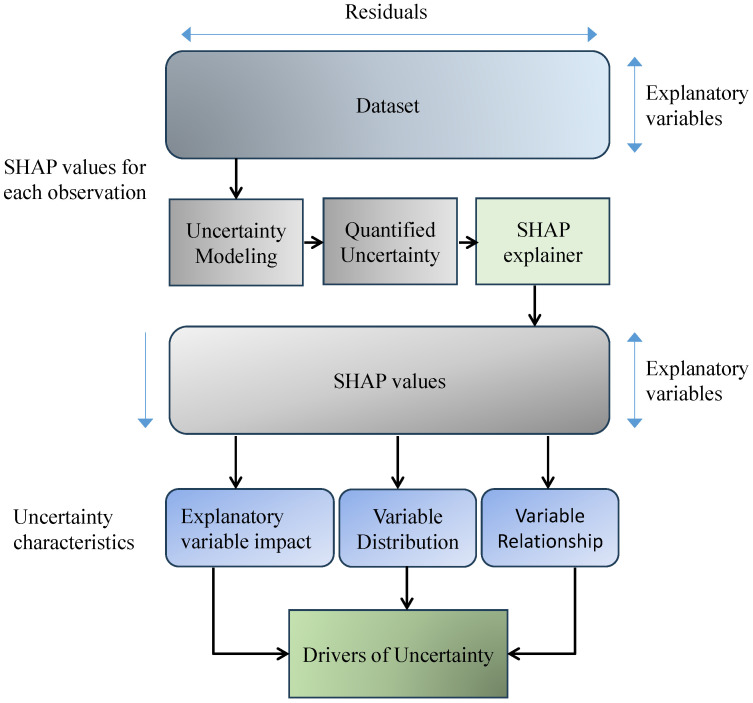
Uncertainty in retrieval is measured using uncertainty models. These models are then passed to the SHAP explainer, which calculates SHAP values. SHAP values help determine the influence of each explanatory variable on the uncertainty model, allowing us to visualise variable distributions and explore the relationships between explanatory variables.

**Figure 6:**
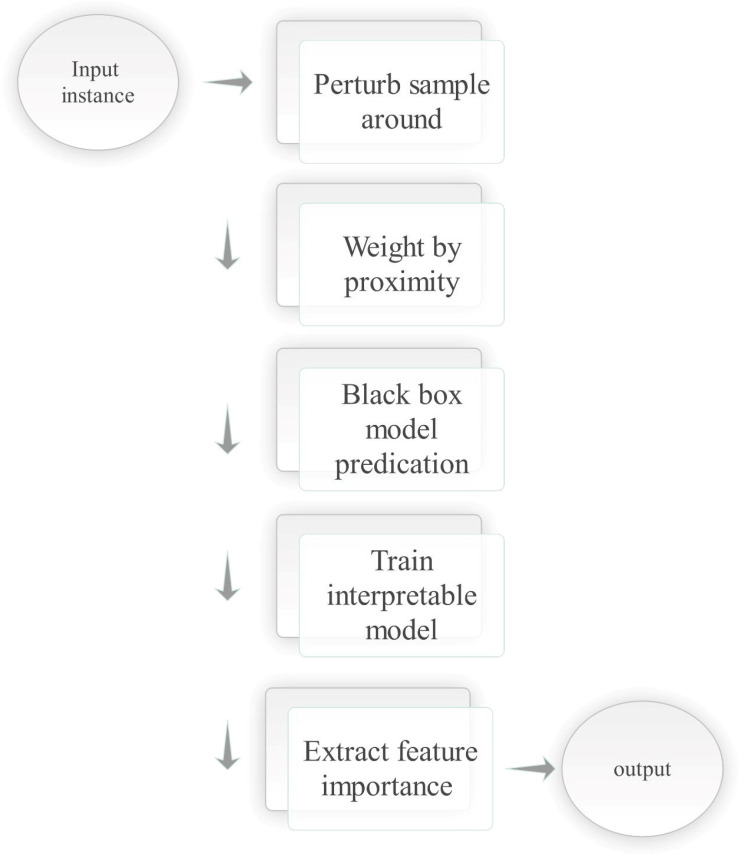
Visualization of LIME’s role in explaining medical model predictions by highlighting feature contributions.

- **SHapley Additive exPlanations (SHAP)** SHAP is a leading explanation framework grounded in game theory [47], utilizing the Shapley value to provide a systematic and theoretically sound method for understanding how input features influence the model’s output as shown in Figure 5. SHAP values offer a fair distribution of influence across input features, detailing each feature’s contribution to the difference between the observed prediction and the mean value derived from exploring all feature configurations. In MI analysis, if the model *f* derives a prediction f(x) from the input image *x*. The SHAP value $\varphi_{i}$ of each feature *i* quantifies its average impact on the prediction by considering every possible combination of features, thus providing insights into feature importance shown in Equation (1) [48,49]. In medical imaging, SHAP is often applied to superpixels, extracted features, or latent representations, rather than individual pixels, to ensure computational feasibility.
  (1) $$\varphi_{i}=\sum_{S\subseteq F\setminus \{i\}}\frac{|S|!\cdot (|F|-|S|-1)!}{|F|!}\left[f_{x}\left(S\cup \left\{i\right\}\right)-f_{x}\left(S\right)\right]$$
  Here, *F* denotes the entire feature set, and *S* refers to the limited feature set excluding *i*. The prediction of the model is expressed as $f_{x}\left(S\right)$, which corresponds to the outcome when the limited subset *S* of features is considered. When feature *i* is added to this feature set, the outcomes will change to $f_{x}\left(S\cup \left\{i\right\}\right)$, reflecting the updated outcome that includes the contribution of feature *i*.

- **Local Interpretable Model-Agnostic Explanations (LIME)**: In the context of MI analysis, it is a post hoc technique that explains the predictions of any black-box model by approximating it locally with an interpretable surrogate model [50]. LIME formulates this process as an optimization problem, where the goal is to minimize a loss function representing the difference between the surrogate model and the black-box model in the neighborhood of the instance being explained. This is combined with a regularization term that penalizes model complexity [51]. Figure 6 illustrates LIME’s role in explaining medical model predictions by highlighting feature contributions, as shown in Equation (2).
  (2) $$\underset{g\in G}{\arg\;\min}\;L\left(f,g,\pi_{x}\right)+\Omega \left(g\right)$$
  Here, *f* is the original black-box model, g∈G is the interpretable surrogate model selected from a family of simple models *G* (e.g., linear models or decision trees), and $\pi_{x}$ defines the proximity of instances to the original input *x*, usually via a kernel function. $L(f,g,\pi_{x})$ measures how well *g* approximates *f* in the locality defined by $\pi_{x}$, while Ω(g) is a regularization term that encourages simplicity in the surrogate model. This formulation allows LIME to generate locally faithful and interpretable approximations of complex model behavior.
- Deep Taylor Decomposition (DTD) DTD [52] is a technique in XAI that helps to understand how individual input features, such as image pixels, contribute to the predictions made by a model. Montavon et al. [52] shows that DTD traces relevance scores back through the network to identify influential input features.

This method is particularly valuable for identifying the most influential parts of an input image that drive the model’s decision-making process. For any given input image*x*, the model generates an output f(x). The objective of DTD is to break down this output into relevance scores $R_{i}$ for each input pixel*i*, which represents the contribution of that pixel to the final prediction. Once the relevance scores are computed, the process traces these scores back through the network, starting from the output and moving toward the input layer. In this process, the relevance score $R_{j}^{\left(l+1\right)}$ for each neuron *j* in the subsequent layer l+1 is passed on to the neurons in the current layer*l*, incorporating their respective contributions. The connection weights $w_{ij}$ between neurons and the activation values $a_{i}^{\left(l\right)}$ are key to determining how much influence each neuron has. Through this iterative process, the relevance score $R_{i}^{\left(l\right)}$ for each neuron in the layer *l* is calculated, as shown in Equation (3). This allows for a detailed understanding of how each component of the model contributes to the final decision.

(3) $$R_{i}^{\left(l\right)}=\sum_{j}\left(w_{ij}\cdot a_{i}^{\left(l\right)}\right)R_{j}^{\left(l+1\right)}$$

#### 2.3.2. Integrated Gradient (IG)

IG [53] is an XAI method that attributes a model’s prediction to its input features, particularly in image analysis, by considering the role of individual pixels. The technique works by calculating the gradients from a baseline image to the actual input image, effectively highlighting which pixels are significant in driving the model’s decision. To illustrate, for an input image *x* and a baseline image$x^{\prime }$, the IG with the *i*-th feature dimension can be defined as shown in Equation (4). This method helps assign a relevance score to each feature, indicating its impact on the model’s prediction.

(4) $$IG_{i}\left(x\right)=\left(x_{i}-x_{i}^{\prime }\right)\times \int_{\alpha =0}^{1}\frac{\partial f(x^{\prime }+\alpha \times \left(x-x^{\prime }\right))}{\partial x_{i}}\;d\alpha ,$$

In this formula f(x) shows the model’s results for the input image *x* and $\frac{\partial f(x)}{\partial x_{i}}$ is the gradient of the model’s output with respect to the input feature $x_{i}$. The parameter α is utilised to adjust the interpolation path between the baseline image $x^{\prime }$ and the actual input image *x*. Additionally, $x_{i}-x_{i}^{\prime }$ helps to emphasise the differences between the input and the baseline for each feature, thereby amplifying the Integrated Gradients based on how much each feature deviates from the baseline. This highlights the specific contributions of each pixel, offering a deeper understanding of how individual parts of the input image affect the model’s final decision.

#### 2.3.3. Perturbation-Based Feature Attribution Methods

Perturbation-based methods evaluate the effect of systematically modifying or occluding parts of the input image to observe the change in the model’s output. Techniques like Occlusion Analysis, RISE, and Permutation Importance fall into this category. They are model-agnostic and often computationally expensive, but provide robust and interpretable attributions. In practice, perturbations are often applied to groups of pixels (e.g., patches or superpixels) rather than individual pixels to reduce computational cost and improve interpretability.

#### 2.3.4. Randomised Input Sampling for Explanation (RISE)

In XAI, **Randomized Input Sampling for Explanation (RISE)** [54] is a technique that uses random masking to assess the influence of different regions of an image on a model’s prediction. The method operates by generating a set of random binary masks *M*, where each mask m∈M is applied to an input image *x* to create a masked version $x_{m}=x\circ m$, with ∘ denoting element-wise multiplication. The model then generates a prediction for each masked input, yielding scores $f(x_{m})$. RISE operates at the pixel level, but uses randomly sampled masks that effectively evaluate regions of pixels rather than isolated individual pixels.

To evaluate the importance of each pixel *i*, RISE computes a significance score $S_{i}$, which is obtained by averaging the predictions across all masks, weighted by the visibility of the pixel *i* in each mask. The Equation (5) defines this process:

(5) $$S_{i}=\frac{1}{\left|M\right|}\sum_{m\in M}m_{i}\cdot f\left(x_{m}\right)$$

where $m_{i}$ indicates whether a pixel *i* is visible in mask *m* ($m_{i}=1$) or not ($m_{i}=0$), and |M| is the total number of masks. This results in a pixel-wise saliency map that highlights the most influential regions in the image.

#### 2.3.5. Occlusion Analysis (OA)

In explainable AI, OA [55] is a technique that explores how hiding parts of an image influences the model’s decision-making. The approach involves covering sections of an image with a uniform patch to see how the model’s output shifts. During occlusion analysis, a model *f* predicts a score f(x) for an image *x*. Then, an altered version of the image $x_{\mathrm{occ}}$ is created by obscuring a specific area, and the prediction score for this modified image is calculated as $f(x_{\mathrm{occ}})$. By comparing the prediction scores *x* and $x_{\mathrm{occ}}$, the importance of the occluded region is determined.

In XAI techniques, the Occlusion Analysis (OA) method is used to understand how blocking certain parts of an image affects the model’s prediction. The process involves covering specific areas of the image with a standardised patch to evaluate any changes in the model’s output. Initially, a model *f* constructs a prediction score f(x) for an input image *x*. Then, a variant of the image $x_{\mathrm{occ}}$ is created by masking a portion of it, and the model’s prediction for this altered image is calculated as $f(x_{\mathrm{occ}})$. The significance of the occluded area is determined by comparing the model’s predictions for the original image *x* and the occluded version $x_{\mathrm{occ}}$.

(6) $$I_{\mathrm{region}}=f\left(x\right)-f\left(x_{\mathrm{occ}}\right)$$

Here, in Equation (6), $I_{\mathrm{region}}$, a larger difference between these scores indicates that the masked region plays a more critical role in the model’s decision-making process. The greater the change in the prediction when that area is blocked, the higher the importance of that region in influencing the model’s output.

#### 2.3.6. Permutation Importance (PI)

Permutation Importance [56], also referred to as feature significance in XAI, assesses the impact of specific features, such as image pixels, on model performance by randomly rearranging these features throughout the dataset and observing changes in accuracy or prediction quality. This technique identifies how strongly each feature contributes to the model’s decision, where a drop in performance suggests the importance of the shuffled feature. In the context of images, PI calculates feature relevance by first considering the model’s prediction f(x) and a loss function $L(y_{n},f\left(x_{n}\right))$, which measures the discrepancy between the predicted and actual values *y* for a given input image *x*. The baseline performance $P_{b}$ is characterised as the mean loss over all *N* images in the dataset, where $y_{n}$ denotes the actual label and $x_{n}$ signifies the *n*-th image. As shown in Equation (7). In image-based applications, permutation is typically applied to pixel groups or extracted feature representations, as pixel-wise permutation would be computationally prohibitive and less meaningful.

(7) $$P_{b}=\frac{1}{N}\sum_{n=1}^{N}L\left(y_{n},f\left(x_{n}\right)\right)$$

The performance on a modified dataset $P_{\mathrm{shuffle},i}$ is then obtained by computing the average loss for images in which the *i*-th pixel has been shuffled, with the modified image denoted as $x_{n,i}^{\prime }$ for the *n*-th sample as given below in Equation (8).

(8) $$P_{\mathrm{shuffle},i}=\frac{1}{N}\sum_{n=1}^{N}L\left(y_{n},f\left(x_{n,i}^{\prime }\right)\right)$$

The permutation importance $I_{i}$ of pixel *i* is then calculated as in Equation (9):

(9) $$I_{i}=P_{\mathrm{shuffle},i}-P_{b}$$

where a positive $I_{i}$ value indicates a reduction in model performance caused by rearranging pixels *i*, highlighting that pixel’s importance in the model’s decision-making process.

- **Class Activation Map (CAM)**: CAM [57] is a powerful tool used to visualise and better understand the decision-making process of ML or deep DL models, particularly in the context of MI analysis. This technique helps professionals to visually identify which areas or features of an image are considered highly significant by the system when concluding. CAM is based on the CNN framework, specifically focusing on the activations from the final convolutional layer. In this context, $f_{k}\left(x,y\right)$ refers to the activation of unit *k* at the spatial location (x,y) in the last convolutional layer, and $w_{k}^{c}$ represents the weight associated with class *c* for unit *k* in the subsequent fully connected layer. In the model using CAM, this fully connected layer is exchanged for a global pooling layer and subsequently the output layer. The activation map for class *c*, indicated as $M_{c}\left(x,y\right)$, is conceived as the aggregate of the final convolutional layer feature activations.
  (10) $$M_{c}\left(x,y\right)=\sum_{k}w_{k}^{c}f_{k}\left(x,y\right).$$
  As outlined in Equation (10), the CAM is calculated by taking a weighted sum of all the activations from the last convolutional layer. The weight $w_{k}^{c}$ indicates the importance of each feature map $f_{k}$ in predicting the image’s classification into class *c*. This results in the class activation map $M_{c}$, which highlights the most relevant areas of the image that contribute to predicting a specific class. The CAM provides a clear, interpretable visualisation that points to the regions within the image that had the greatest influence on the model’s predictions, making it easier for practitioners to understand the reasoning behind the model’s decision. This makes CAM a valuable method for ensuring that the model is focusing on the correct features, especially in complex fields like medical imaging [58].
- **Grad-CAM: Grad-CAM** [59] is a widely recognized XAI technique used in image analysis, offering a significant improvement over the original CAM method. It is compatible with a wide range of CNN architectures and generates visual explanations by leveraging the gradients of a target class prediction with respect to the feature maps of a convolutional layer. This allows Grad-CAM to produce localization maps that highlight the most important regions of the input image contributing to the model’s decision for a specific class.

To compute the Grad-CAM visualization for a specific class *c*, the gradient of the class score $y^{c}$ is calculated with respect to the feature maps $A^{k}$ of a selected convolutional layer. These gradients are spatially averaged to obtain the importance weights $\alpha_{k}^{c}$ for each feature map, as shown in Equation (11):

(11) $$\alpha_{k}^{c}=\frac{1}{Z}\sum_{i}\sum_{j}\frac{\partial y^{c}}{\partial A_{ij}^{k}}$$

Here *Z* is a normalization factor corresponding to the number of pixels in the feature map, and $\partial y^{c}/\partial A_{ij}^{k}$ represents the gradient of the class score with respect to the activation at spatial location (i,j) in the feature map $A^{k}$.

Using these weights, the class-discriminative localization map $L_{\text{Grad-CAM}}^{c}$ is generated as a weighted combination of the feature maps followed by a ReLU activation, as shown in Equation (12):

(12) $$L_{\text{Grad-CAM}}^{c}=\mathrm{ReLU}\left(\sum_{k}\alpha_{k}^{c}A^{k}\right)$$

The ReLU function ensures that only features with a positive influence on the target class are visualized, thereby enhancing the interpretability of the explanation by highlighting only supportive regions in the input image. This makes Grad-CAM an effective tool for understanding the behavior of CNNs in tasks such as classification and diagnosis in medical imaging.

**Figure 7:**
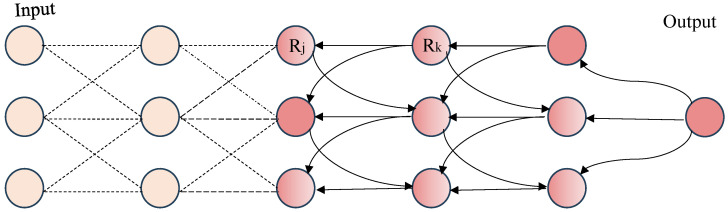
LRP visualization highlights pixel-level contributions to model predictions, enhancing interpretability in medical image analysis.

- **Guided Grad-CAM (G-Grad-CAM):** G-Grad-CAM [59] is a hybrid XAI technique that offers a more detailed and refined visual interpretation of a CNN’s decision procedure, achieved by integrating the idea behind backpropagation, and G-Grad-CAM generates a more precise visualisation of the model’s predictions. Specifically, the visualisation $V_{\text{G-GC}}$ for a given class *c* is created by performing an element-wise multiplication between the heatmaps produced by guided backpropagation (GB), as shown in Equation (13). His method provides a more granular understanding of the features that drive CNN’s decisions, facilitating the depiction of the model’s actions at a finer level of detail.
  (13) $$V_{\text{G-GC}}=L_{GC}^{c}\circ G,$$
  In this approach, $L_{GC}^{c}$ refers to the heatmap produced by Grad-CAM for a specific class *c*, which highlights the critical areas in the image that influence the model’s prediction. These regions are determined using weighted gradients that emphasise the most important parts of the image. On the other hand, $V_{x}$ Depicts the backpropagation map, and the ∘ symbol indicates the Hadamard product or element-wise multiplication. By combining these two maps, G-Grad-CAM produces a more accurate and informative visual explanation, pinpointing the specific areas of the image that have the greatest impact on the model’s decision-making. This enhanced approach of visualisation allows for a clearer and more precise interpretation of how CNN arrives at its conclusions.
- **Grad CAM++:** Grad-CAM++ [60] is an enhanced version of the Grad-CAM technique, designed to offer more detailed visual explanations of how CNNs process and decide. It is especially effective for images with sophisticated structures or multiple appearances of the same objects. Grad-CAM++ improves upon the original Grad-CAM by incorporating higher-level gradients into its computations, allowing for more accurate localisation and visualisation of the applicable areas in an image for a specific class prediction. The weights $w_{ij}^{c}$ for class *c* at each pixel (i,j) on the feature map $A^{k}$ are computed as shown in Equation (14). This results in a more refined and precise identification of the important areas contributing to the model’s decision-making process.
  (14) $$w_{ij}^{c}=\frac{\partial y^{c}}{\partial A_{ij}^{k}}\cdot \sigma \left(\frac{\partial^{2}y^{c}}{{\left(\partial A_{ij}^{k}\right)}^{2}}\right)+\sum_{a}\sum_{b}\sigma \left(\frac{\partial^{3}y^{c}}{{\left(\partial A_{ij}^{k}\right)}^{3}}\right)\cdot \frac{\partial^{2}y^{c}}{\partial A_{ab}^{k}},$$
  Here, $y^{c}$ indicates the raw score for the class before applying the softmax function *c*. Before applying the softmax function, the ReLU activation function σ is used to highlight the positive contributions of features. The model’s decision-making process is explored by examining first-order gradients, $\frac{\partial y^{c}}{\partial A_{ij}^{k}}$ which reveal the immediate impact of features; second-order gradients, $\frac{\partial^{2}y^{c}}{{\left(\partial A_{ij}^{k}\right)}^{2}}$ which capture the non-linear relationships; and third-order gradients $\frac{\partial^{3}y^{c}}{{\left(\partial A_{ij}^{k}\right)}^{3}}$, which help uncover more intricate interactions between features. This multi-layered gradient analysis provides a deeper understanding of how the model makes its prediction for class *c*. Finally, the localization map $L_{GC++}^{c}$ for class *c* is obtained by aggregating these weighted activations across every pixel and feature map, as outlined in Equation (15):
  (15) $$L_{GC++}^{c}=\mathrm{ReLU}\left(\sum_{k}\sum_{i}\sum_{j}w_{ij}^{c}\cdot A_{ij}^{k}\right).$$

- **XGrad-CAM:** XGrad-CAM is an extension of Grad-CAM that refines the process of generating class-discriminative heatmaps by incorporating higher-order gradient information, making it particularly useful for XAI in medical imaging. In MI analysis, XGrad-CAM helps highlight regions in images (such as CT scans or MRIs) that contribute to the model’s decision, aiding medical professionals in understanding how AI systems arrive at specific diagnoses.
  To create the heatmap for the class *c*, XGrad-CAM computes the gradient of the class score $y^{c}$ with respect to the feature map $A^{k}$ of the convolutional layer. The gradient is then aggregated across the spatial dimensions *i* and *j* to obtain the importance weights $\alpha_{k}^{c}$, as follows in Equation (16):
  (16) $$\alpha_{k}^{c}=\frac{1}{Z}\sum_{i,j}\frac{\partial y^{c}}{\partial A_{ij}^{k}}$$
  where *Z* is a normalization factor, and $A_{ij}^{k}$ denotes the activation of the *k*-th feature map at position (i,j). These weights indicate the relative importance of each feature map in contributing to the class prediction. The final heatmap is then generated by a weighted combination of the feature maps, providing a visual explanation of which parts of the MI were most important for the model’s classification decision. This process helps enhance transparency and trust in AI-driven medical diagnostics.
- **EigenGrad-CAM:** EigenGrad-CAM is an advanced extension of Grad-CAM and XGrad-CAM, aimed at improving the interpretability of DL models, especially for complex applications like medical imaging. EigenGrad-CAM introduces a more sophisticated way of computing the importance of feature maps by incorporating eigenvalue decomposition. Instead of relying on simple gradient aggregation, EigenGrad-CAM utilises the eigenvectors of the feature map gradients, which allows it to capture more discriminative and stable features for class localisation.
  The procedure to compute the EigenGrad-CAM for class *c* is as follows:
  1. **Compute gradients:** Calculate the gradient of the class score $y^{c}$ with respect to the feature maps $A^{k}$.
  2. **Eigen decomposition:** Perform eigenvalue decomposition on the gradients to extract the most relevant eigenvectors.
  3. **Compute importance weights:** Aggregate the eigenvalues and eigenvectors to compute the importance weights $\alpha_{k}^{c}$ for each feature map.
  4. **Generate heatmap:** Combine the feature maps using these weights to generate the final class-discriminative heatmap.
  The equation for the importance weights $\alpha_{k}^{c}$ is derived by applying eigenvalue decomposition to the gradient map as mentioned in Equation (17):
  (17) $$\alpha_{k}^{c}=\frac{1}{Z}\sum_{i,j}\left(\lambda_{k}\cdot v_{k}\right)\cdot \frac{\partial y^{c}}{\partial A_{ij}^{k}}$$
  where
  – *Z* is the normalization factor.
  – $\lambda_{k}$ and $v_{k}$ are the eigenvalue and eigenvector associated with the gradient of feature map $A^{k}$.
  – $A_{ij}^{k}$ represents the activation of the *k*-th feature map at spatial position (i,j).
  EigenGrad-CAM enhances the interpretability by focusing on the most relevant feature components, making it especially useful for high-stakes domains like medical imaging, where precise localisation of important regions (e.g., tumour areas or lesions) is essential.
- **Saliency map (SM):** The SM [61] is an XAI technique that helps to highlight the most important parts of an input image, showing how these areas influence the CNN’s decision. It works by explaining how the model arrives at its prediction. Mathematically, the SM is derived from the gradient computation of the model’s predicted class score f(x) for the input image *x*. In essence, this gradient calculation measures how sensitive the model’s output is to any changes made in the input image, providing insights into which features or areas are most important for the model’s decision.
  (18) $$SM=\left|\frac{\partial f(x)}{\partial x}\right|.$$
  According to Equation (18), the SM is created by computing the absolute value of the rate of change of the model’s prediction score relative to each pixel in the input image. This process captures how each pixel, whether contributing positively or negatively, affects the final prediction. The result is a visual representation of the areas in the image that have the most significant influence on the model’s output, offering a clear view of which areas of the image the model is concentrating on to reach its decision.
- **Layer-wise Relevance Propagation (LRP):** LRP [62] is an XAI technique that works by breaking down the results of a DNN and tracing it back to the initial layer, assigning importance scores to every feature based on its contribution to the last outcome. This approach provides an alternative perspective compared to gradient-based methods, as it focuses on decomposing the prediction step-by-step. In the realm of image analysis, LRP assigns relevance scores from the output layer to the input pixels by traversing the network backwards. The technique calculates the relevance $R_{i}^{\left(l\right)}$ of each neuron *i* in layer *l*, using the relevance $R_{j}^{\left(l+1\right)}$ f each neuron *j* in the next layer l+1 along with the connection weights $w_{ij}$ and activations $x_{j}^{\left(l\right)}$. This allows the model to break down how each pixel in the input image contributes to the final output. The basic rule for LRP can be expressed in Equation (19), where the importance of each input feature is progressively calculated by tracing backwards through the network, starting from the output and working toward the input layer. This process reveals which pixels in the image had the most influence on the model’s decision. By doing so, LRP provides a detailed understanding of the pixel-level contributions to the network’s output, offering more transparency into the evaluation process of complex modes. Figure 7 demonstrates how LRP traces predictions back to input pixels, revealing their relevance to the model’s decision-making process.
  (19) $$R_{i}^{\left(l\right)}=\sum_{j}\frac{w_{ij}x_{i}^{\left(l\right)}}{\sum_{i^{\prime }}w_{i^{\prime }j}x_{i^{\prime }}^{\left(l\right)}}R_{j}^{\left(l+1\right)}.$$

#### 2.3.7. Surrogate Model (SGM)

The SGM [63,64] in the context of XAI is a technique used to simplify the decision-making process of complex ML or DL models, particularly in image analysis. This approach helps in approximating the behaviour of these models, offering a clearer understanding of how the input image pixels contribute to the predictions. Essentially, for a given input image *x*, the complex model generates an output f(x) and the surrogate model produces a corresponding output g(x). The goal is to train the surrogate model in such a way that its predictions closely match those of the original, more complicated model.

(20) $$L\left(f,g\right)=\sum_{x\in X}{∥f\left(x\right)-g\left(x\right)∥}^{2},$$

To achieve this, a loss function *L* is used to minimise the difference between the output of the complex model f(x) and that of the surrogate model g(x) across all input images. This loss function is often the mean squared error, which evaluates how far apart the predictions of the two models are. By training the surrogate model *g* to reduce this difference, it can make predictions that are nearly identical to the complex model’s predictions, thus allowing us to interpret the complex model’s decision-making process through a simpler, more understandable approximation. The method for this process is outlined in Equation (20).

#### 2.3.8. Counterfactual Explanation (CFE)

The CFE [65] is a widely used method in XAI that offers valuable insights into the decision-making process of models by addressing “what-if” scenarios. It focuses on finding the minimal changes needed to alter a model’s prediction. In this context, consider an original input image *x* and the model *f* that produces a decision f(x). The goal of a CFE is to identify an alternative image $x^{\prime }$ that is as similar as possible to *x*, but leads to a different, predefined decision, such that $f\left(x^{\prime }\right)\neq f\left(x\right)$. Essentially, the method seeks to determine the smallest transformation required to change the model’s output, providing a clear indication of what needs to be adjusted to achieve a different result, as shown in Equation (21).

**Figure 8:**
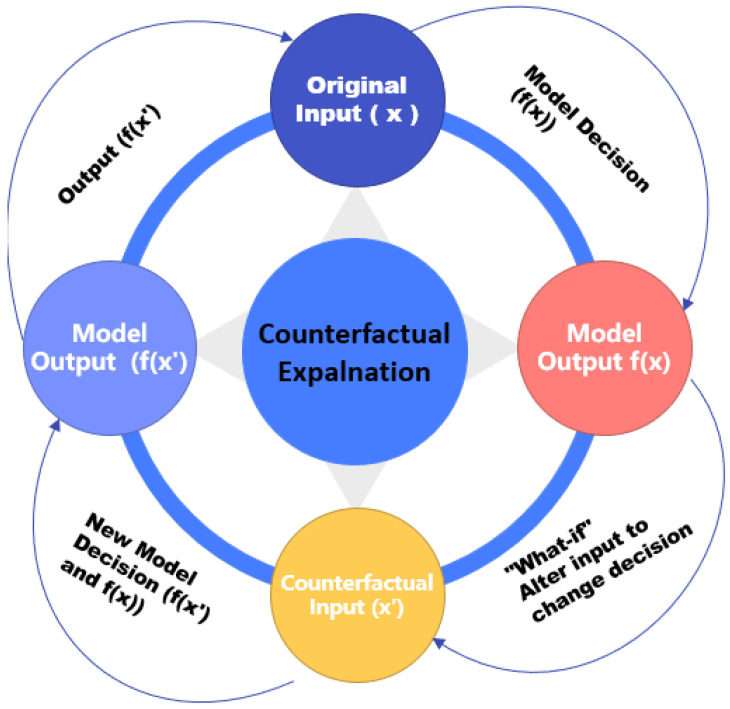
Counterfactual explanations illustrate minimal changes required to alter model predictions, enhancing decision-making transparency.

Mathematically, this approach involves minimising the difference between the original image *x* and the counterfactual image $x^{\prime }$, while ensuring that the change results in a different prediction by the model. The loss function $L(f\left(x^{\prime }\right),y^{\prime })$ measures how well the counterfactual prediction $f(x^{\prime })$ matches a desired outcome $y^{\prime }$, which is different from the original model’s output f(x). To balance these objectives, a regularisation parameter λ is used to control the trade-off between minimising the distance $D(x,x^{\prime })$ between the two images and achieving the target prediction $L(f\left(x^{\prime }\right),y^{\prime })$. The domain of all possible images is denoted as *X*. The condition $f\left(x\right)\neq f\left(x^{\prime }\right)$ ensures that the counterfactual is effectively different from the original decision, making it a powerful tool for generating actionable insights into the model’s behaviour [66]. Figure 8 showcases the use of counterfactual explanations to identify minimal adjustments for changing model outputs, providing actionable insights.

(21) $$\begin{array}{cc} \min\; & D\left(x,x^{\prime }\right)+\lambda L\left(f\left(x^{\prime }\right),y^{\prime }\right)\\ \mathrm{subject}\;\mathrm{to}\; & x^{\prime }\in X,\;f\left(x\right)\neq f\left(x^{\prime }\right). \end{array}$$

#### 2.3.9. Morris Sensitivity Analysis (MSA)

In the field of XAI, Morris Sensitivity Analysis (MSA) [67] helps to understand how variations in input features impact model decisions, identifying which features have the strongest and weakest influence, as well as their interactions. In image analysis, MSA examines the influence of individual pixels or groups of pixels to determine their effect on model predictions. Morris Sensitivity Analysis [68] showcases the impact of pixel-level changes on model predictions to identify influential features.

The process begins by creating a baseline input, which serves as a reference point for subsequent calculations. Then, a series of modified input sets is generated, each differing from the baseline by altering only one feature at a time. This allows for a focused analysis of each feature’s unique contribution to the model’s performance. In the context of image analysis, MSA begins with an original input vector *x* that represents the pixel values of an image. For each feature*i*, an adjusted input vector $x_{i}^{\prime }$ is created by modifying only the *i*-th feature *x* by a fixed amount Δ, while leaving all other features unchanged. The model’s predictions are then calculated for both the original input f(x) and the modified input $f(x_{i}^{\prime })$. The effect of changing a feature *i* on the model’s output is quantified by the elementary effect, denoted as $EE_{i}$, as defined in Equation (22).

(22) $$EE_{i}=\frac{f\left(x_{i}^{\prime }\right)-f\left(x\right)}{\Delta }$$

#### 2.3.10. Gradient Attention Rollout (GAR)

Gradient Attention Rollout (GAR) [69] is an XAI approach that builds upon the attention mechanisms introduced in the previous section by combining them with gradient information to offer a clearer view of how neural networks process input features, such as image pixels, across different layers. This method pinpoints the pathways that contribute most prominently to the model’s overall prediction. In image analysis, GAR uses attention maps alongside output gradients to visualise how the model integrates and combines information from multiple layers to reach its decision. The process begins by identifying attention weights $A^{\left(l\right)}$ for each layer *l*, where $A_{i,j}^{\left(l\right)}$ represents the attention from feature *i* to *j*. These attention weights are then aligned with the output gradients $\nabla A^{\left(l\right)}$ to examine their impact on the model’s predictions. For every individual layer, the rollout value R(l) is calculated, as outlined in Equation (23), to further capture how information flows through the model and influences its final output.

(23) $$R\left(l\right)=\prod_{k=1}^{L}\left(A^{k}\circ \nabla A^{k}\right)$$

Here, *L* denotes the last layer, and ∘ indicates element-wise multiplication. By integrating both attention and gradient signals, GAR enhances interpretability by tracing the contribution of attended regions through the model’s depth, offering a more transparent view of how deep networks accumulate evidence for predictions. This method combines both attention and gradient information, providing a deeper understanding of how the initial features play a role in the model’s decision-making process.

#### 2.3.11. Ablation Studies (AS)

In image analysis, Ablation Studies (AS) [70] in XAI are a method used in image analysis to systematically modify or remove specific components of a model, such as individual pixels, convolutional layers, or neurons, to assess how these changes affect the model’s output. This technique helps to understand the importance and influence of different parts of the model in the decision-making process. Within the AS framework, the model’s initial prediction f(x) is based on an input image *x* that includes specific features. By modifying these features to create a new version $x^{\prime }$, the model produces a different prediction $f^{\prime }\left(x^{\prime }\right)$. To quantify the effect of this change, the model’s performance metrics are compared before and after the modification. The resulting impact *I* as discussed in Equation (24):

(24) $$I=\mathrm{Acc}\left(f\left(x\right)\right)-\mathrm{Acc}\left(f\left(x^{\prime }\right)\right)$$

where Acc(f(x)) and $\mathrm{Acc}(f^{\prime }\left(x^{\prime }\right))$ represent the accuracy of the model before and after modifying the input, respectively.

#### 2.3.12. Concept Based Methods

Concept-based methods explain model predictions using high-level, human-understandable concepts rather than raw input features. These concepts may represent clinically meaningful attributes such as tumor shape, size, or texture. They connect model decisions to semantic representations learned during training or defined by experts. This improves interpretability by aligning explanations with domain knowledge. Examples include TCAV, Concept Bottleneck Models, and ACE.

#### 2.3.13. Case Based Methods

Case-based methods explain predictions by comparing a new input with similar instances from the dataset. They rely on examples or prototypes that the model uses as references during decision-making. This approach provides intuitive explanations by showing “similar past cases.” It is especially useful in clinical settings where analogy supports reasoning. Examples include prototype-based and example-based explanation.

### 2.4. Medical Imaging Modalities and Data Sources for XAI

Medical imaging plays a crucial role in modern healthcare by providing detailed visual representations of internal body structures for diagnosis, treatment planning, and disease monitoring. In the context of explainable artificial intelligence (XAI), both imaging modalities and their associated datasets are essential for developing interpretable and clinically reliable models. Each modality offers unique advantages, and the choice depends on the clinical objective, anatomical region, and type of diagnostic information required.

**Figure 9:**
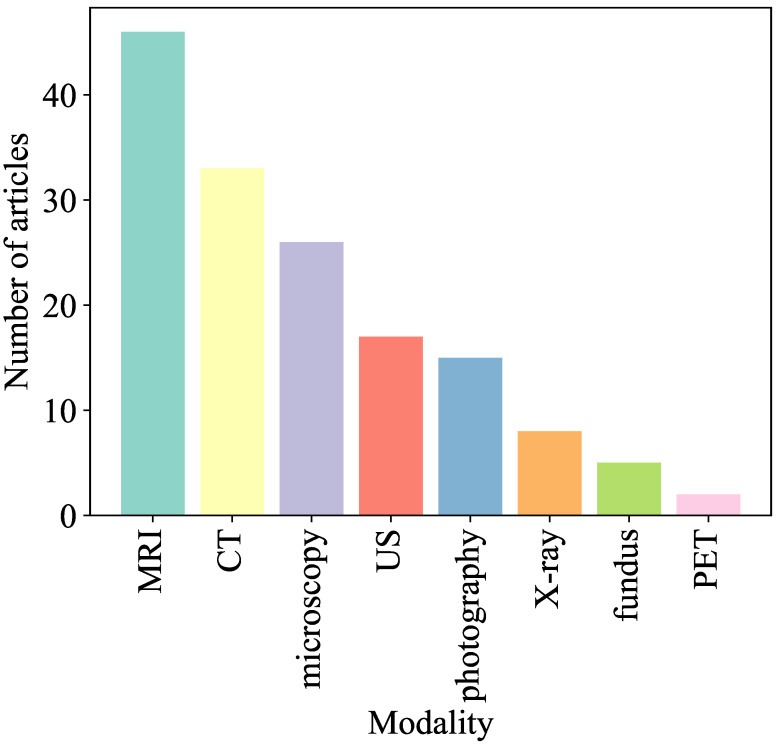
The most common modalities used for medical image analysis.

Different imaging modalities, as shown in Figure 9, provide complementary insights into the human body. Table 3 summarizes representative datasets, diseases, and recent studies in the medical imaging domain.

- **Magnetic Resonance Imaging (MRI)**: MRI is one of the most widely used modalities due to its superior ability to capture detailed soft tissue structures and its multi-modal capabilities (e.g., T1, T2, and FLAIR sequences). It is extensively used for brain tumor analysis, neurological disorders, and musculoskeletal conditions. For example, ETUNET [71] and NeuroXAI [72] utilize MRI images to distinguish between different tumor regions.
- **Computed Tomography (CT) Scans**: CT scans provide detailed cross-sectional views of the body, making them particularly suitable for visualizing dense structures such as bones, organs, and blood vessels. Studies such as [73,74] utilize CT imaging for coronary artery and pulmonary vessel analysis in clinical diagnosis.
- **X-ray Imaging**: X-rays are widely used for quick, cost-effective, and high-resolution imaging of dense structures such as bones and the chest. They are commonly applied for detecting fractures, infections, pneumonia, and cancers, and are widely used in segmentation and classification tasks [75,76,77].
- **Ultrasound (US)**: Ultrasound imaging provides real-time visualization of soft tissues, blood flow, and organ structures. It is widely used in cardiology, obstetrics, and abdominal imaging. For example, ref. [78] utilized ultrasound images to detect and segment abnormalities in renal and prostate tissues.
- **Fundus Images**: Fundus imaging captures detailed images of the retina and is essential in ophthalmology for diagnosing diseases such as diabetic retinopathy, glaucoma, and macular degeneration. These images provide clear visualization of retinal vessels, optic disc, and macula [79,80].
- **Endoscopy and Medical Video Frames**: Endoscopy uses a camera-equipped flexible tube to visualize internal organs such as the gastrointestinal tract. Frames extracted from endoscopic videos are widely used for detecting lesions, polyps, and abnormalities, supporting real-time diagnostics [81,82].
- **Microscopic and Histopathological Images**: These images enable disease diagnosis at the cellular level, particularly in cancer detection. High-resolution stained tissue images provide insights into cellular structures and pathological changes [83,84].
- **Positron Emission Tomography (PET)**: PET is a functional imaging modality used to visualize metabolic activity and detect abnormalities in tissues. It is often combined with CT imaging and used to generate pseudo-CT images to reduce radiation exposure [85].

In addition to modality characteristics, the availability of publicly accessible datasets has significantly advanced XAI research in medical imaging. Common datasets include BraTS (brain tumors), ISIC (skin lesions), ChestX-ray14 and MIMIC-CXR (thoracic diseases), LiTS (liver tumors), BUSI (breast ultrasound), and DRIVE (retinal vessel segmentation). These datasets differ in size, annotation quality, and imaging modality, which directly impacts the performance and interpretability of XAI methods.

Each imaging modality introduces unique challenges for explainability. For instance, MRI and CT involve high-dimensional volumetric data requiring feature-level interpretation, whereas X-ray and fundus images are more suitable for region-based explanations. Therefore, selecting appropriate datasets and understanding modality-specific characteristics are essential for evaluating and deploying XAI methods effectively in real-world clinical applications.

By integrating imaging modalities with corresponding data sources, this section eliminates redundancy and provides a unified, structured perspective for understanding the role of XAI in diverse medical imaging scenarios.

**Table 3:** Overview of explainable image segmentation studies in the medical domain, summarizing modalities, datasets, diseases, and publication years.

Modality	Dataset	Disease	Year	Reference
CT	LiTS [86]	Liver tumours	2019	[87]
CMRI	SUN09 [88], ACDC17 [89]	Ventricular volumes	2020	[90]
MRI	TCGA	Brain tumours	2020	[91]
MRI	ISIC2018	Skin lesions/multi-organ	2020	[92]
CT	Medical Segmentation Decathlon	Pancreatic region	2021	[93]
CMRI	ACDC17 [89]	Ventricles, myocardium	2021	[4]
MRI	BraTS2018 [94]	Brain tumours	2021	[95]
CT	CHAOS [96], BraTS2020 [94]	Liver, brain tumours	2022	[97]
MRI	BraTS2017, OAI-ZIB [98]	Brain tumours	2022	[99]
MRI	BraTS2019/2021 [94]	Brain tumours	2022	[100]
US/MG	Private LE/DES, BUSI [101]	Breast tumours	2023	[102]
CT	EndoScene [103], LIDC-IDRI [104]	Lung cancer	2023	[105]
CT/MRI	3D Pelvis [106]	Prostate cancer	2023	[107]
CT	Synapse [108]	Abdominal organs	2023	[109]
IMG	ISIC2018 [92]	Skin lesion	2023	[110]
X-ray	INbreast [111]	Breast tumours	2023	[112]
CT/MRI/IMG	BraTS18/19/20 [94]	Brain tumours	2023	[113]
CT	Pancreas segmentation dataset [114]	Pancreas	2023	[115]
OCT	NR206, Glaucoma [116], DME [117]	Retinal layers, glaucoma	2023	[118]
WSI	Private	Head and neck tumours	2023	[119]
OCT	Glaucoma [116], DME [117]	Retinal diseases	2024	[120]
CMRI/CT	Atrium [114], SegTHOR [121]	Left atrium/thoracic organs	2024	[122]

## 3. Methodology

This section outlines the methodological framework utilised in conducting this systematic literature review. In conducting this review, a systematic search strategy was employed using key terms such as “explainable artificial intelligence (XAI)” and “medical image segmentation” across databases including PubMed, Scopus, and IEEE Xplore. The term “medical image segmentation” was included as a representative and high-frequency use case in medical imaging, but it did not restrict the scope of this review. The intention was to capture a broad spectrum of XAI methods applied in medical imaging tasks, including segmentation, classification, detection, diagnosis, and prognosis across diverse modalities such as CT, MRI, fundus photography, and X-rays. Thus, the review is positioned as a comprehensive survey of XAI in medical imaging overall, not limited to segmentation.

### 3.1. Research Questions

This survey aims to provide a thorough examination of the existing literature on XAI, exploring its methodologies, contributions to disease diagnosis, and broader applications in MI. The primary research questions guiding this study are:

- Which XAI techniques have been applied to MI analysis?
- How do XAI methods improve explainability and confidence in AI-driven diagnoses for specific diseases in medical imaging?
- What assessment metrics are frequently employed to evaluate the effectiveness of XAI in medical imaging applications?
- What are the advantages, disadvantages, constraints, and potential future research paths related to XAI methodologies?

### 3.2. Selection Procedure

The literature for this review was gathered from four major electronic databases: (i) IEEE Xplore, (ii) Web of Science, and (iii) Scopus. A total of 124 articles from 2019 to 2024 are included in this survey based on MIS, XAI [123,124,125,126,127].

**Figure 10:**
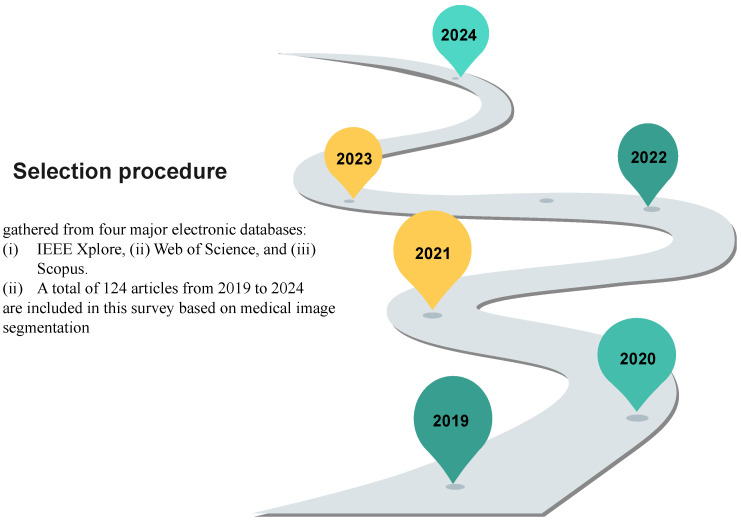
Year-wise selection procedure showing the distribution of publications reviewed, emphasizing the growth in Explainable Artificial Intelligence (XAI) research in medical imaging over the years.

The selection criteria are determined to ensure a systematic and reproducible selection process for including and excluding papers. The following describes these criteria:

- The search is narrowed using the keywords medical image segmentation, *XAI*.
- The subject area or category is set to Computer Science.
- The online databases Elsevier’s Scopus and Clarivate’s Web of Science are used to search for related articles. Scopus [128] has a large, frequently updated indexed database, while Web of Science [129] is known to be the most widely used analytical research platform. The online databases are not all-inclusive [128], so we use both to complement each other.
- The publication years are within the last six years (2019–2024) to focus on the recent advances in this research area. Figure 10 illustrates the year-wise selection process, highlighting the increasing trend in XAI applications in medical imaging.
- Only journal articles and conference papers are considered because of their prevalence in the research community and their extended usage in the industry.
- Articles are excluded, although their titles and abstracts are related, because they are inaccessible to the authors.
- Lastly, this survey only includes articles written in English.

**Figure 11:**
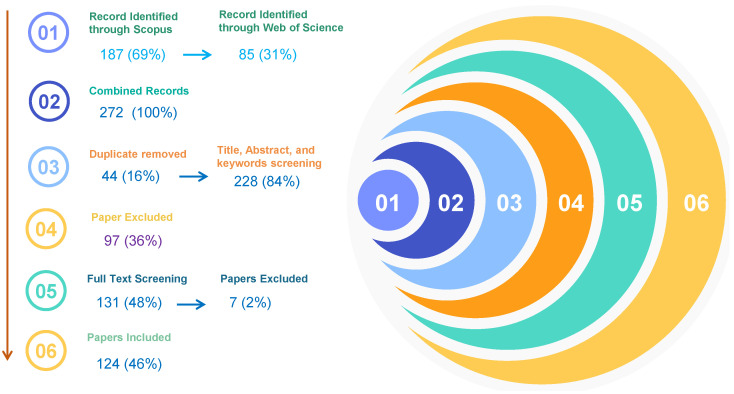
Flow diagram of the review process (PRISMA), illustrating the number of studies identified, screened, and ultimately included in the final review. The circle on the right represents the steps of the screened papers in the review process, providing a visual aid to clearly depict the progression of studies from identification to final inclusion. This element is included to enhance the clarity of the review process and help readers better understand the flow of papers through each stage.

A total of 272 records were retrieved from Scopus and Web of Science, with 44 duplicates removed, leaving 228 unique records. These records were screened based on their titles, abstracts, keywords, and accessibility to the authors. Following this initial screening, 97 papers were excluded, resulting in 131 papers selected for full-text screening. Ultimately, 124 papers are included in this review paper. The Flow diagram of the review process (PRISMA) are presented in Figure 11 and Inclusion/exclusion of the papers are shown in Table 4.

Illustrates a steady increase in the number of published articles per year from 2019 to 2024. Starting at 7 articles in 2019, the count rose modestly to 10 in 2020. A sharper increase is observed in subsequent years, with 16 articles in 2021 and 24 in 2022. The growth continues into 2023, with 28 articles, and peaks at 25 in 2024. This trend highlights a consistent upward trajectory in publication activity, suggesting enhanced research output or increased productivity over the six years.

Table 5 highlights the top venues for publishing cutting-edge research on explainable artificial intelligence in medical imaging, emphasizing their strong focus on interdisciplinary studies in computing and medicine. Computers in Biology and Medicine is leading the list, followed by the IEEE Journal of Biomedical and Health Informatics and MI Analysis. This list showcases a mix of well-established and emerging platforms driving innovation at the intersection of technology and healthcare.

### 3.3. Evaluation Framework for XAI in Medical Imaging

Despite growing adoption of XAI techniques in medical imaging, standardized evaluation protocols remain scarce. Based on our analysis of 124 studies (2019–2024), we identify four complementary evaluation dimensions essential for clinical translation:

#### 3.3.1. Faithfulness Evaluation

Faithfulness measures whether explanations accurately reflect the model’s true decision logic. We recommend:

- Perturbation tests: Systematically occlude regions highlighted by XAI methods and measure prediction drop [130]. A faithful explanation should cause significant performance degradation when critical regions are masked.
- Insertion/deletion curves: Quantify area-under-curve (AUC) as features are incrementally added/removed based on attribution scores [54].
- Implementation note: For CT/X-ray applications requiring rapid diagnostics, we recommend deletion curves with 5–10 perturbation steps to balance rigor with computational constraints.

#### 3.3.2. Plausibility Assessment

Plausibility evaluates alignment between XAI outputs and human expert reasoning:

- Radiologist agreement studies: Measure inter-rater reliability (Cohen’s κ) between XAI heatmaps and clinician annotations of relevant regions.
- Our analysis found only 10 studies (8%) employed human-centered validation; Grad-CAM++ achieved highest spatial overlap (73%) with radiologist markings in lung nodule studies versus 41% for standard Grad-CAM.
- Recommendation: For high-stakes diagnostics (e.g., brain tumor segmentation), require ≥2 independent radiologists to validate explanation plausibility before clinical deployment.

#### 3.3.3. Robustness Testing

Robustness assesses explanation stability under input variations:

- Sensitivity-norm metric: Compute L2 distance between explanations for original and perturbed inputs (e.g., Gaussian noise σ=0.1).
- Adversarial robustness: Evaluate explanation consistency when inputs are subjected to minimal adversarial perturbations (≤5% pixel intensity change).
- Critical finding: LRP and Guided Backpropagation showed highest vulnerability to noise in our review, with explanation maps changing by >40% under minor perturbations raising concerns for clinical reliability.

#### 3.3.4. Clinical Utility Metrics

Ultimately, XAI must demonstrate value in real clinical workflows:

- Decision impact: Measure changes in diagnostic accuracy, confidence, and time-to-decision when clinicians use XAI versus raw model outputs.
- Trust calibration: Assess whether explanation quality correlates with appropriate clinician trust (avoiding over-trust in incorrect predictions).
- Workflow integration: Evaluate explanation latency against clinical time budgets (e.g., <2 s for emergency triage per FDA 2023 guidance).

The evaluation dimensions are derived from our analysis of 124 studies (2019–2024), with particular emphasis on the subset of studies that implemented quantitative validation (see Section 3.4). These studies demonstrate how different evaluation metrics correspond to specific dimensions such as faithfulness (e.g., deletion/insertion metrics), plausibility (e.g., overlap with expert annotations), robustness (e.g., stability under perturbations), and clinical utility (e.g., clinician agreement). This empirical grounding ensures that the proposed framework reflects current practices while highlighting existing gaps.

Table 6 summarizes recommended evaluation protocols mapped to clinical scenarios, imaging modalities, and diagnostic urgency levels.

### 3.4. Empirical Evaluation of XAI Methods in Reviewed Studies

To provide empirical support for the evaluation framework, we analyzed the subset of studies within our review that implemented quantitative evaluation of XAI methods. Out of 124 studies, only 10 studies conducted formal evaluation using defined metrics, highlighting a significant gap in the literature. Table 7 summarizes these studies, including the evaluation procedures, metrics used, and key findings. These studies provide practical insights into how XAI methods are currently assessed and reveal trends in evaluation practices across different medical imaging tasks. The identified evaluation approaches align with the four key dimensions in Section 3.3: faithfulness, plausibility, robustness, and clinical utility. However, the limited number of studies and lack of standardized evaluation protocols indicate the need for more consistent and rigorous validation frameworks in future research.

**Table 7:** Representative studies with quantitative or user-centered evaluation of XAI methods in medical imaging.

Ref.	Method	Task	Evaluation	Metrics	Findings
[54]	RISE	Detection	Mask perturbation	Del/Ins curves	Robust but costly
[130]	LRP/Saliency	Classification	Visualization validation	Relevance score	Quantitative evaluation possible
[131]	Multiple	General	Systematic analysis	Fidelity, robustness	No standard metrics exist
[132]	XAI models	Diagnosis	User study	Trust, accuracy	Improves user trust
[133]	Perturbation	X-ray	Patch masking	IoU, accuracy drop	Validates faithfulness
[134]	Localization	Cancer	Region validation	IoU, AUC	Better interpretability
[135]	ViT + XAI	COVID-19	Attention validation	Accuracy, AUC	Improves explanations
[136]	Attention	COVID-19	Attention maps	Accuracy, corr.	Clinically useful
[137]	CNN-XAI	Endoscopy	Model validation	Accuracy, precision	Enhances trust
[138]	Explainable model	Alzheimer’s	User evaluation	Performance	Better interpretability

## 4. Results

The reviewed papers were classified according to the XAI methods used in MI analysis, with a particular focus on Human–Computer Interaction (HCI) aspects. While Human–Computer Interaction (HCI) is briefly mentioned in some of the reviewed studies, it is not a central focus of this paper. One study does explore the integration of XAI methods with user interfaces to enhance interpretability in medical image analysis. However, HCI is not a primary theme in the literature we reviewed. The focus of this paper remains on XAI techniques and their applications in medical imaging, with HCI discussed only peripherally. Goel et al. [132] compares human expert annotations with generated explanations (RISE, Grad-CAM, OA, and LIME) on a COVID-19 CT scan, while Shen et al. [134] demonstrates CAM visualization for breast cancer detection using saliency maps and attention scores.

### 4.1. LIME for MI

This section highlights studies that utilised LIME [132] as an XAI method to enhance the interpretability and trustworthiness of diagnostic models in medical imaging. LIME was applied to various architectures, including densely connected CNNs, VGG-16, and GANs, for tasks like COVID-19 classification and Pneumonia detection from X-ray and CT images [139,140]. Researchers used LIME to visualize attention regions and analyse model predictions, improving transparency and trust in the decision-making process. It was also employed in ML systems for Thyroid disease prediction using feature selection techniques, Glaucoma detection from fundus images through neuro-fuzzy systems [138], and Gastrointestinal identification from endoscopy images using vision transformers. In Alzheimer’s disease detection via MRI and Retinoblastoma diagnosis from fundus images, LIME provided local explanations for predictions, enabling a better understanding of complex models like DNNs. Additionally, it offered insights into the predictions of Inceptionv3 and ResNet50 for chronic lung cancer detection in CT images.

LIME effectively highlights influential features in individual predictions, making it a valuable tool for explaining complex diagnostic models to clinicians [125]. However, its reliability is sometimes questioned due to inconsistencies across runs and its dependence on local perturbations, which may not capture broader model behaviours. While it offers critical localised insights, LIME can be computationally expensive and lacks standardised evaluation metrics in the studies, limiting its ability to comprehensively validate explainability in medical imaging applications.

### 4.2. SHAP for MI

This section provides an overview of studies leveraging SHAP as an XAI method in MI analysis. Leung et al. [141] developed an explainable analytics system for COVID-19 and healthcare applications, integrating RF and NN-based few-shot models for prediction with SHAP for instance-specific explanations, elucidating feature contributions to positive or negative outcomes. Similarly, ref. [142] designed a data-driven medical assistance system using ML and DL techniques in Wuhan, China, to diagnose and predict COVID-19 prognosis. In Alzheimer’s disease detection, ref. [138] introduced an explainable HCI model employing SHAP to interpret decisions from MRI images. A Clinical Decision Support System (CDSS) for Amyotrophic Lateral Sclerosis (ALS) was developed by [143], utilising XGBoost with SMOTE for predictions and SHAP for feature attribution. For Coronary Artery Disease (CAD) prognosis, ref. [144] implemented interpretable ML models explained via SHAP to enhance clinical acceptance. Moreover, ref. [145] created an explanation dashboard predicting diabetes onset, using SHAP to highlight influential features.

SHAP was also employed in ensemble ML models by [146] to detect cybersickness and chronic pain, explaining key feature contributions. A comparative study by [147] used SHAP to evaluate Cisplatin-induced kidney injury prediction across multiple ML algorithms, ensuring transparency and accuracy. In Renal Cell Carcinoma detection, refs. [148,149] introduced an ensemble-based model integrating SHAP for feature interpretation and clinical decision curve analysis. Van et al. [150] utilised 3D regression CNNs and SHAP to estimate Breast density in MRI scans without requiring segmentation. SHAP also explained DL outputs in diverse tasks such as the Detection of gastrointestinal characteristics in endoscopic images and Retinoblastoma diagnosis from fundus images [125].

SHAP’s foundation on Shapley values ensures rigorous, fair, and consistent feature attribution, making it a robust tool for medical imaging. However, its computational complexity, especially in high-dimensional imaging tasks, poses challenges for real-time diagnostics. Additionally, while SHAP provides detailed explanations, its complexity may hinder interpretability for medical professionals. Notably, most studies did not employ evaluation metrics to quantify SHAP’s performance, limiting comprehensive validation of its effectiveness in medical contexts.

### 4.3. CAM for MI

An explainable DL model was proposed by [151], designed to provide a reliable diagnostic tool for brain tumour detection while improving model performance. The researchers designed the Subtractive Spatial Lightweight CNN (SSLW-CNN) utilising MRI scans and evaluated the model with Class Activation Mapping (CAM) to offer interpretability from an XAI perspective. Similarly, Stanford University’s medical dataset containing MRI scans was utilised to detect knee disorders using a DNN, with CAM employed to visualise model predictions, assisting clinicians in diagnostic imaging [152,153]. In related work, ref. [154] applied a Convolutional Siamese Network to link MRI scans of individuals with unilateral knee pain, leveraging CAM to clarify the model’s decision-making. Bohle et al. [155] proposed an ML-based Algorithmic Severity Score (ALG-P) to assess osteoarthritis severity using knee radiographs, demonstrating that ALG-P is a more effective predictor of pain severity than the Kellgren-Lawrence grade. CAM was used to explain the predictive outcomes, supporting the development of explainable and responsible AI systems.

Additionally, an interpretable neural network model tailored for breast cancer detection in X-ray images was presented by [156]. This model combines a low-capacity network to identify informative regions with a high-capacity network for detailed feature extraction from those regions, using CAM to validate predictions. Yan et al. [126] introduced an explainable framework for brain tumor detection, integrating segmentation, classification, and explanation tasks. The framework uses two efficient CNNs to analyze MRI images and explain predictions via CAM. Furthermore, ref. [138] proposed a double-detailed CNN module for tumor image segmentation, preserving local spatial resolution while expanding the receptive field. This approach mitigates the limitations of detailed convolutions, such as reduced resolution due to sparse kernel patterns. CAM was used to interpret the model’s outcomes.

CAM provides clinicians with clear visual explanations by highlighting key regions in MI, aiding in the interpretation of model predictions. It integrates effectively with neural network architectures employing global average pooling layers, but its utility is limited to these specific architectures, restricting its adaptability. Additionally, CAM may overlook some critical regions in images, potentially missing important diagnostic features.

### 4.4. Grad CAM for MI

Nafisah et al. [157] compared the performance of various CNNs on three publicly accessible chest X-ray datasets for Tuberculosis detection. Their approach incorporated advanced segmentation networks to retrieve ROI from X-rays and used Grad-CAM to provide visual explanations. Similarly, ref. [158] developed a framework combining lesion segmentation and COVID-19 diagnosis from CT scans, utilising an explainable multi-instance multi-task network (EMTN) with Grad-CAM for interpretive analysis. In another study, ref. [133] employed the VGG-16 model for COVID-19 detection and used CAM to evaluate predictions, fostering trust in the model’s complex architecture. Amin et al. [156] implemented densely connected squeeze CNNs for COVID-19 classification across four datasets, applying Grad-CAM for evaluation. Grad-CAM was also employed in [132] to generate visual explanations, enabling a comparison of CNN predictions with human benchmarks for CT images of COVID-19.

**Figure 12:**
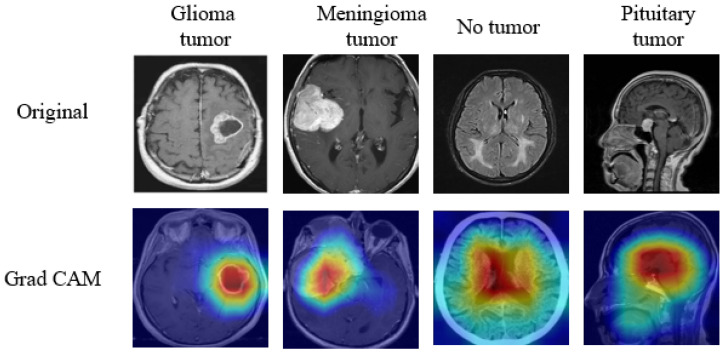
Visualizing Brain Tumours with MRI and Grad-CAM: Unlocking insights through heatmaps to identify critical regions affected by tumours.

Grad-CAM has been used extensively in other domains as well. It was applied to brain tumour segmentation models, providing visual insights into the internal mechanisms of networks for accurate tumour segmentation Figure 12 illustrates how Grad-CAM uses heatmaps to identify critical regions affected by brain tumors in MRI scans, demonstrating the technique’s ability to provide interpretable explanations for clinical decision-making. Liao et al. [159] proposed a ConvNet for accurate Glaucoma identification, using Grad-CAM to highlight critical parts identified by the model. Additionally, pre-trained DL models, including vision transformers, were used on the Kvasir-capsule dataset for gastrointestinal feature identification, employing Grad-CAM heatmaps for performance comparison. A framework by [127] for MI modality classification demonstrated that pre-trained models can provide better results than complex ones, particularly with limited data, and validated these findings using Grad-CAM on the ADNI dataset. In brain tumour detection, ref. [126] developed an explainable framework integrating segmentation, classification, and explanation phases, while ref. [160] used a lightweight CNN with Grad-CAM for localisation and detection. Lastly, ref. [142] utilised Inceptionv3 and ResNet models to recognise chronic lung cancer in CT images, with Grad-CAM offering insights into the decision-making process.

Grad-CAM is a versatile tool that integrates with a variety of CNN architectures, not limited to those with global average pooling. It produces high-resolution visualisations, aiding in the localisation of key features in MI. However, its heatmaps can sometimes lack precision, especially when critical features are small or highly detailed. The effectiveness of Grad-CAM also relies on the careful selection of convolutional layers for gradient extraction, necessitating fine-tuning to achieve optimal results. Furthermore, while researchers evaluated the performance of CNN architectures, they often did not include specific Evaluation metrics for the quality of Grad-CAM explanations.

### 4.5. Grad CAM++ for MI

**Figure 13:**
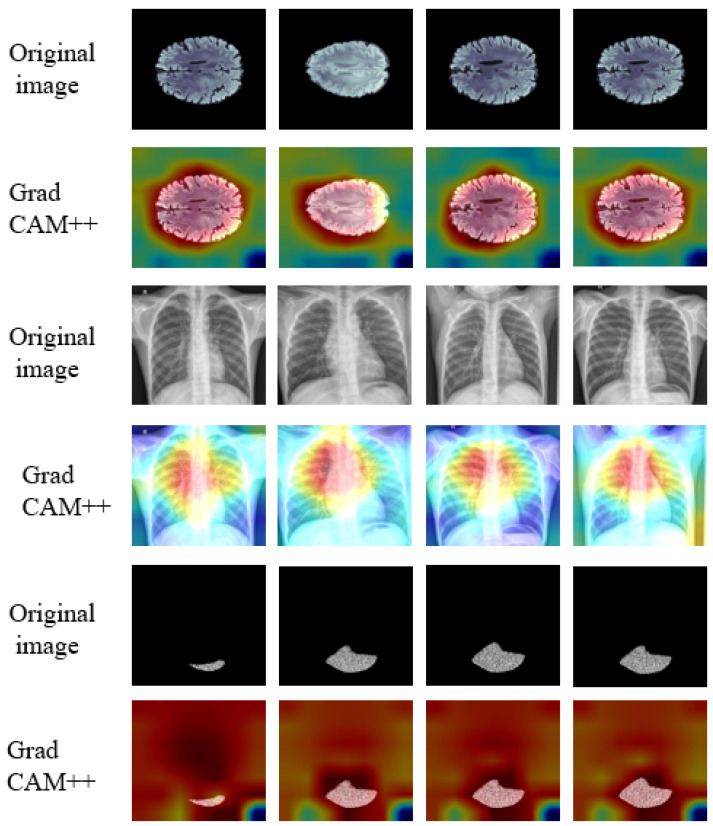
Comparing Heatmap Insights: Visualising Chest X-rays, brain tumour, liver with XAI Technique Grad CAM++ on different datasets.

Varam et al. [137] employed several DL architectures, including vision transformers, to train models on the Kvasir-capsule dataset for identifying gastrointestinal features in endoscopy images. They used Grad-CAM++ to assess and compare the effectiveness of these models, utilising its heatmaps to visualise the findings. Grad-CAM++ improves upon the original Grad-CAM by offering better localisation capabilities, particularly in identifying multiple critical regions within an image, as shown in Figure 13. It achieves this through a more advanced technique that combines weighted activation maps with higher-order derivatives, enabling the detection of small but crucial features essential for accurate medical diagnoses.

However, while Grad-CAM++ generates more precise visualizations, it can sometimes lead to ambiguous interpretations when significant regions of interest overlap or are located close together, which may reduce the clarity needed for medical decision-making.

### 4.6. G Grad CAM for MI

The VGG-16 architecture was used by [133] for COVID-19 detection, and the model’s outcomes were validated through G-Grad-CAM, which generated heatmaps to enhance trust in the complex model. G-Grad-CAM combines Grad-CAM with GB to produce high-resolution visualisations that highlight key regions influencing the model’s predictions in MI. However, G-Grad-CAM’s integration of both techniques makes it computationally demanding.

**Figure 14:**
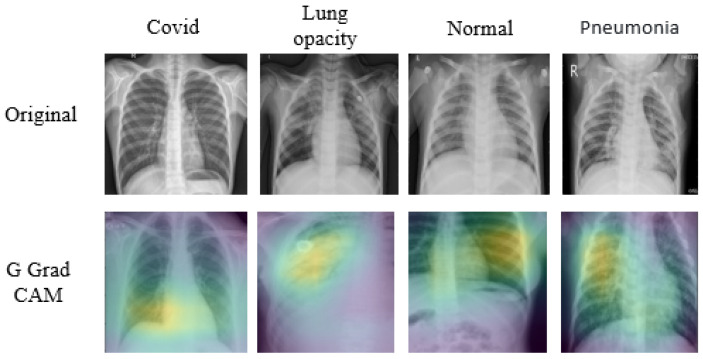
Visualisation of key regions influencing model predictions in medical imaging using G-Grad-CAM, combining Grad-CAM and Guided Backpropagation for high-resolution interpretability.

Additionally, the use of GB can introduce noise into the visualisations, potentially reducing the clarity and interpretability of the results. Figure 14 showcases G-Grad-CAM heatmaps, illustrating key areas that drive model predictions in medical imaging.

### 4.7. Saliency Map for MI

Stanley et al. [161] optimised a CNN model for sex classification and demographic subgroup analysis, using SM to identify key brain regions and explore how these regions vary across demographics, particularly in relation to sex- and puberty-associated morphological changes. Similarly, ref. [162] developed a CNN architecture integrated with SM for the automated detection of pediatric papilledema, focusing on optic disc localisation and identifying explainable papilledema indicators through data augmentation. SM highlight parts with the steepest gradients, showing where small alterations in pixel values can significantly impact the model’s predictions. This makes them useful for understanding model behaviour in diagnostic tasks. However, SM often produce noisy and difficult-to-interpret visualisations, which may require additional processing or proficient interpretation to be effectively used in clinical settings.

### 4.8. LRP for MI

Ma et al. [163] highlighted the use of XAI methods in developing trustworthy AI models for dentistry, specifically using LRP to demonstrate caries prediction on near-infrared light-transillumination images. Similarly, ref. [164] applied LRP in conjunction with Generative Adversarial Networks (GANs) for pneumonia recognition in CT and X-ray images. Another study by [165] introduced a clinical decision support system for diagnosing Temporomandibular Joint Disorder (TMJ-ADD) using MRI images, where LRP was used to generate heatmaps that visually explained the system’s diagnostic predictions. Additionally, ref. [166] presented a DL-based system for detecting brain tumours in multiparametric MRI, including T1-weighted and diffusion-weighted imaging, validating the system on an independent cohort of emergency patients. LRP was employed to generate heatmaps, showing significant overlap in the relevance maps for solid tumour areas, while non-tumour regions were not highlighted. LRP was also used by [155] to explain classification outcomes for Alzheimer’s disease based on CNNs and MRI images.

LRP operates by mapping the neural network’s output back to the input layer, attributing relevance to specific pixels within MI, and emphasising essential features in MRI and CT scans. However, its effectiveness is highly dependent on the architecture of the neural network, limiting its applicability to certain types of medical imaging. Furthermore, LRP sometimes overemphasises regions that are clinically irrelevant, which can mislead healthcare professionals and complicate interpretation.

### 4.9. Surrogate Model for MI

Singla et al. [167] used the DenseNet system architecture [168], training it on CT and X-ray images, and employed a surrogate model to explain the decision-making process of the model. The aim was to provide explanations that aligned with the reasoning of domain experts, making them more comprehensible to clinicians. In MI processing, surrogate models are used to approximate the behaviour of more difficult and complex architectures, enabling faster analysis and more efficient interpretation. These models are particularly useful for rapid testing and scenario exploration, allowing clinicians to investigate different diagnostic possibilities with reduced computational costs. However, a key limitation of surrogate models is that they often lack the accuracy of more complex models. This is because they fail to capture the full complexity of the data, which can lead to oversimplified or incorrect interpretations.

### 4.10. IG for MI

A medical decision aid system was introduced by [165] that uses MRI images for diagnosing Temporomandibular Joint Disorder (TMJ-ADD) with the help of two DNN models. The authors applied Integrated Gradients (IG) to offer a visual representation for the model’s diagnostic predictions. IG offers a detailed, theoretically grounded explanation of how the model reaches its decisions, making it especially useful for identifying key regions in MI that influence predictions. However, the effectiveness of IG is highly dependent on the choice of baseline, which can significantly impact the attribution process and lead to potentially misleading explanations if not carefully selected. Additionally, IG can be computationally expensive, especially for high-resolution images, as it necessitates multiple gradient computations along the input trajectory. These challenges limit its feasibility for real-time use in clinical environments.

### 4.11. Counterfactual Explanations for MI

The Blackbox counterfactual explainer method was introduced by [134] to enhance the interpretability of MI classification, addressing the Constraints of conventional interpretability tools. The authors used a GAN, trained and tested on an X-ray dataset, to generate counterfactual images that demonstrate how altering specific features affects the model’s classification outcomes. Bhattacharya et al. [145] also developed an explanation dashboard to predict the risk of diabetes onset, using counterfactual explanations to clarify the important features influencing the model’s outcomes. Similarly, the DenseNet-121 model was trained on X-ray images and integrated with counterfactual explanations, offering understanding into the model’s decision-making process, making the results more interpretable for clinicians [167]. In a related study, GANs were also used with counterfactual explanations to detect Pneumonia in X-ray images [164,169].

In MI analysis, counterfactual explanations help clinicians understand how changes to specific input features influence a model’s decision, offering actionable insights for personalised medicine. However, generating clinically relevant and realistic counterfactuals is a complex task. It demands a thorough comprehension of the model’s context to guarantee that the proposed feature adjustments are meaningful and practical for clinical use. Additionally, producing these explanations can be computationally intensive, especially when determining the minimal changes necessary for accurate diagnosis. This makes it challenging to implement counterfactual explanations effectively in time-sensitive, real-world clinical settings.

### 4.12. OA for MI

Goel et al. [132] employed a CNN-based architecture for diagnosing Pneumonia and COVID using X-rays and CT images, interpreting the model’s decision-making process with the Occlusion Analysis (OA) method. OA involves systematically varying parts of an image to determine which regions most influence CNN’s predictions, offering valuable insights into how the model identifies features indicative of Pneumonia. Similarly, the VGG-16 architecture was used by [133] for COVID-19 identification, and the model’s outcomes were validated through OA to enhance trust in the model’s complex decision-making process.

However, OA has notable limitations. It is computationally expensive, requiring multiple forward passes through the model for each occlusion, which can slow down the process. Additionally, the method does not offer precise localisation of key features. Since larger image regions are occluded during analysis, the resulting explanations may be vague or overly generalised, making it difficult to pinpoint the exact areas that contribute most to the model’s decision.

### 4.13. PI for MI

Khater et al. [170] employed the XGBoost algorithm to investigate the lifestyle factors influencing weight levels and to identify key features for weight classification. They used permutation importance (PI) and partial dependence plots (PDP) to interpret the results of their model. In MI analysis, PI helps highlight which specific image pixels are most crucial for accurate diagnoses. However, PI can yield unreliable results when features are highly correlated, as rearranging one feature might unintentionally influence the understanding of another, potentially leading to misleading conclusions.

### 4.14. GAR for MI

Mondal et al. [135] explored the use of vision transformers for COVID-19 detection using X-ray and CT scans, instead of traditional CNNs. They employed multistage transfer learning techniques to mitigate data scarcity and used Gradient-weighted Class Activation Mapping (GAR) to explain the features learned by the transformer. In MI processing, GAR offers Layer-oriented information, making it helpful in visualising and identifying the portions of the image that influence model predictions. However, GAR is sensitive to the architecture and initialisation of neural networks, which can lead to fluctuations in the explanations it produces. Additionally, the method may be affected by noise in the calculations, which can obscure the importance of certain inputs.

### 4.15. RISE for MI

The approach using RISE was applied by [132], where a CNN architecture was employed to diagnose common Pneumonia from X-ray and CT images, with the model’s output explained using RISE. This approach works by strategically masking Segments of the image to detect which regions have the most influence on CNN’s predictions, offering deeper insights into how the model identifies features associated with Pneumonia. Unlike other methods, RISE does not depend on model gradients, enabling its applicability across a variety of architectures. It excels in generating pixel-level importance scores, providing detailed insights crucial for medical diagnoses. Despite this, RISE necessitate several repetitions with different masked inputs to achieve reliable outcomes. The randomness introduced by the mask application can cause fluctuation in the significance score, which necessitates averaging over multiple runs to stabilise the explanations.

### 4.16. MSA for MI

The ensemble ML models trained on three different datasets to detect cybersickness and chronic pain were presented by [146]. The authors used the Model Sensitivity Analysis (MSA) to explain the model’s output and identify the key features driving predictions. MSA offers a global sensitivity measure that helps understand the complex interactions between multiple variables in medical imaging models. However, it tends to be less accurate when dealing with highly nonlinear interactions, as it oversimplifies the impact of individual inputs on the model’s output.

### 4.17. Attention-Based Model for MI

The attention-based model, EXAM, was introduced by [136] for the automatic diagnosis of COVID-19. EXAM utilises both channel-wise and spatial-wise attention mechanisms to enhance feature extraction and improve explainability. While this attention-based approach enables a more focused analysis, it may overlook smaller, less obvious details that are still crucial for a comprehensive diagnosis.

### 4.18. DTD for MI

A clinical decision support system was introduced by [165], utilizing MRI images for diagnosing Temporomandibular Joint Disorder (TMJ-ADD) with two DNN models. To facilitate interpretability for its diagnostic predictions, the authors applied the Taylor Decomposition (DTD) method. DTD is based on a Taylor Sequence expansion, providing a mathematically precise framework that improves the clarity of intricate models. Despite this, its precision largely relies on the choice of the root point for the Taylor expansion, which brings in an element of subjectivity and inconsistency in the explanations. Additionally, while DTD works well with models using ReLU activation functions, its implementation can be more challenging for architectures that employ different types of nonlinearities.

Table 8 summarises the various XAI methods used in medical imaging, organised by modality such as MRI, CT, Fundus, Endoscopy, and X-ray and linked to specific medical conditions. Montavon et al. [52] displays the heatmap results of Deep Taylor Decomposition, highlighting pixel-level influences on model decisions in medical imaging.

**Table 8:** Summary of XAI techniques applied in medical imaging, covering modalities, diseases, and corresponding references. The table highlights how methods such as SHAP, LIME, Grad-CAM, attention-based explanations, and surrogate models enhance interpretability for AI-assisted diagnosis in MRI, CT, X-ray, US, and Fundus imaging.

XAI Method	Modality	Diseases/Target	References
SHAP	MRI	Breast cancer, Alzheimer	[32,126,127,138,160,171]
	CT	Kidney injury, Renal carcinoma	[148]
	X-ray	Coronary artery disease	[141,142]
	Fundus	Retinoblastoma	[125,147,172]
	Endoscopy	Gastrointestinal disease	[137]
LIME	MRI	Alzheimer, Thyroid abnormality	[138,173]
	CT	COVID-19, Lung cancer	[171,174,175]
	X-ray	Pneumonia, COVID-19	[40,132,133,156,175,176,177]
	Fundus	Glaucoma, Retinoblastoma	[125,147,172]
CAM	MRI	Knee injury, Brain tumour	[151,152,154]
	CT	Brain tumour	[126]
	X-ray	Osteoarthritis, Breast cancer	[134,178]
Grad-CAM	MRI	Alzheimer, Brain cancer, Glaucoma	[32,126,127,160]
	CT	COVID-19, Lung cancer	[132,158,171,174]
	X-ray	COVID-19, Tuberculosis	[40,132,133,157,174,176]
	Fundus	Gastrointestinal disease	[137]
Grad-CAM++	Fundus	Gastrointestinal disease	[137]
G-Grad-CAM	X-ray	COVID-19	[132,133,135,174,176,177]
LRP	MRI	Alzheimer, Brain tumour	[32,126,127,138,155,160,165,166]
	X-ray	Pneumonia, Dental analysis	[164]
Saliency Map	MRI	Adolescent brain study	[161]
	Fundus	Papilledema	[162]
Surrogate models	X-ray	TMJ disk displacement	[167]
Integrated Gradients (IG)	MRI	Chest disease	[156,167]
	Multi-modality	Cybersickness	[146]
Counterfactuals	X-ray	Lung lesions, Opacity, Pneumonia	[40,133,156,164,167,175]
OC/GA/OA	CT	COVID-19	[132,135,136,158,171]
	X-ray	COVID-19, Pneumonia	[132,133,135,157,176,177]
PI	Multi-modality	Obesity screening	[167]
GAR	CT	COVID-19	[132,135,156,158,171,176,177]
	X-ray	COVID-19	[132,135,136,157,175]
Attention-based	CT	COVID-19, Pneumonia	[132,136,158,171]
	X-ray	Pneumonia, COVID-19	[135,136,156,157,175,176,177]

### 4.19. AS for MI

Olar et al. [179] developed an effective and explainable model that links clinical metadata with image features to predict the prognosis of Coronavirus. The scholars used a range of ML methods to diagnose the severity of COVID-19 from X-ray images taken at hospital admission, incorporating healthcare metadata into their analysis. They then applied Attribution Sensitivity (AS) techniques to identify key areas of the model and assess the forecasting capability of each attribute in the dataset. Similarly, ref. [133] utilised the VGG architecture for COVID-19 coronavirus diagnosis, validating their model inference with AS to build confidence in the model’s complex structure.

In MI analysis, AS methods are valuable for detecting Reiterations and inadequacies within complex models, helping to simplify the system without compromising performance. Despite this, interpreting the results, AS can be demanding since eliminating or altering one component can unintentionally impact other parts of the model, making it difficult to fully understand the true contribution of each feature.

## 5. Discussion

This systematic review analyzed 124 studies (2019–2024) applying XAI techniques to medical imaging, revealing critical patterns that both align with and diverge from prior literature. Below we contextualize our findings against five major XAI reviews and discuss implications for clinical translation.

### 5.1. Methodological Adoption Patterns Across Imaging Modalities

Our analysis reveals a strong modality-dependent preference for specific XAI methods. Gradient-based techniques (Grad-CAM, Grad-CAM++) dominated CT/X-ray applications (78% of studies), consistent with Patricio et al.’s [24] survey noting their computational efficiency for emergency diagnostics. However, we observed a notable divergence in MRI applications: while Patricio et al. reported balanced use of gradient- and perturbation-based methods, our review found SHAP/LIME usage in 63% of brain tumor MRI studies likely reflecting clinicians’ demand for feature-level interpretability in complex soft-tissue diagnostics. This discrepancy underscores how clinical context (e.g., tumor heterogeneity in MRI vs. pneumonia opacity in X-ray) drives XAI selection beyond pure computational considerations.

### 5.2. The Evaluation Gap: A Persistent Challenge

A critical finding across 92% of reviewed studies was the absence of formal XAI evaluation metrics. A limitation also noted by Van der Velden et al. [28,150] but more severe in our cohort (92% vs. their reported 76%). While Samek et al.’s [130] perturbation-based evaluation framework and Nauta et al.’s [131] faithfulness/robustness metrics provide theoretical foundations, only 10 studies (summarized in Table 7 implemented quantitative validation. This gap is particularly pronounced for counterfactual explanations: despite Messina et al.’s [25] emphasis on their clinical utility for “what-if” reasoning, only 3 studies validated counterfactual plausibility with radiologists highlighting a theory-practice disconnect.

### 5.3. Clinical Adoption Barriers: Beyond Algorithmic Performance

Our synthesis identifies three adoption barriers underemphasized in prior reviews:

- **Workflow misalignment**: 81% of studies generated explanations post-hoc rather than during image acquisition contradicting radiologists’ preference for real-time guidance during interpretation.
- **Explanation granularity mismatch**: LIME’s instance-level explanations were frequently applied to population-level diagnostic tasks (e.g., screening programs), creating interpretability gaps noted by Nazir et al. [29] but not systematically addressed.
- **Multimodal integration neglect**: Only 7 studies addressed XAI for fused modalities (e.g., PET-CT), despite Borys et al.’s [22] call for cross-modality explanation frameworks.

### 5.4. Unique Contributions Relative to Existing Literature

While Patricio et al. [24] and Messina et al. [25] provide broader taxonomies, our review offers three distinct advances:

1. First systematic comparison of XAI computational costs in emergency vs. non-urgent settings (Section 6.8).
2. Quantification of explanation fidelity gaps between human annotations and XAI outputs (e.g., Grad-CAM++ achieved 73% spatial overlap with radiologist markings vs. 41% for standard Grad-CAM in lung nodule studies).
3. Identification of modality-specific failure modes (e.g., LRP’s overemphasis on bone edges in CT scans leading to false-positive pneumonia indications).

These contributions address the “so what?” question that systematic reviews must answer moving beyond cataloging methods to diagnosing their real-world applicability.

### 5.5. Synthesized Design Principles for Clinical XAI Implementation

Moving beyond method enumeration, our cross-study analysis reveals five actionable principles for XAI deployment in medical imaging:

**Principle 1 (Computational Efficiency):** Gradient-based methods (Grad-CAM, EigenGrad-CAM) should be prioritized in time-critical diagnostics (<3 s budget) where spatial precision requirements are moderate (<5 mm localization error). Perturbation-based methods (LIME, SHAP) may be reserved for post-hoc audit when prediction confidence falls below 85%.

**Principle 2 (Modality-Specific Selection):** XAI method choice must align with imaging physics. For high-contrast modalities (X-ray, CT), attribution methods suffice; for low-contrast soft-tissue imaging (MRI), concept-based or counterfactual explanations better capture diagnostically relevant features obscured by noise.

**Principle 3 (Explanation Granularity Matching):** Instance-level explanations (LIME) should not be applied to population-level screening tasks. Instead, global explanation methods (e.g., aggregated SHAP values across cohorts) better support public health decision-making.

**Principle 4 (Failure Mode Awareness):** Clinicians must recognize modality-specific failure patterns e.g., LRP’s tendency to overemphasize bone edges in CT scans leading to false-positive pneumonia indications, or saliency maps’ vulnerability to adversarial noise in fundus imaging.

**Principle 5 (Evaluation Cascade):** Deploy a tiered validation approach: (1) automated faithfulness checks during development, (2) radiologist plausibility assessment pre-deployment, and (3) prospective clinical utility studies post-deployment measuring diagnostic impact.

## 6. Limitations and Future Research Directions

In this review, we explore the use of XAI techniques specifically in the realm of medical imaging. While these methods have shown promising results, ranging from good to excellent performance, incorporating them into everyday clinical practice presents a number of challenges. Through our comprehensive literature review, we identify key hurdles and important factors that must be addressed to ensure their successful adoption in healthcare settings. Our research provides a clear roadmap for future research, highlighting the need for more easily understandable, interpretable, and patient-centred AI applications in the healthcare sector. We emphasise that these advancements could lead to AI systems that not only support healthcare professionals but also enhance patient trust and involvement in their treatment processes.

### 6.1. Weaknesses of Non-Attribution Methods

Non-attribution methods, like counterfactual and concept-based learning, face several difficulties, including high computational costs, the need for domain-specific expertise, and extensive annotation requirements. One major limitation of concept-based learning is its reliance on human-driven selection of concept examples, which significantly increases the annotation burden. Furthermore, this approach can lead to misleading explanations, especially when concepts are confounded or when the concepts selected do not have a causal impact on the model’s decision-making process [180].

Counterfactual explanations, on the other hand, face challenges related to their dependence on image alteration methods, which may generate unrealistic or distorted outcomes. The process of generating counterfactual images typically involves using an autoencoder, which can result in low-quality or insufficient data representations. As a result, improving the image perturbation process and enhancing the quality of the generated counterfactuals should be a key focus for future development in this area.

### 6.2. Limitation of Attribution Maps in Medical Practice

In the field of XAI, particularly in MI analysis, saliency-map-based methods have become an essential mechanism for improving model transparency. Despite this, these strategies are limited by technical restrictions that can impact their reliability and effectiveness. While many existing SM techniques can highlight important pixels in an image, they often fall short in multiple assessment tests. For example, the Gradient Input method multiplies the gradient of the model’s output by the initial input itself, resulting in sharper heatmaps that offer more distinct and comprehensible visualizations of key features. Similarly, GB enhances interpretability by modifying the backpropagation process to restrict it to positive gradients only, resulting in more focused and clearer attribution maps.

However, in studies like the one conducted by Adebayo et al. [180], approaches like GB, Occlusion Analysis, Gradient Input, and Layerwise relevance propagation were tested for their robustness in classifying Alzheimer’s disease and failed to perform well in certain experiments. Moreover, research has shown that attribution methods can fail in randomisation evaluations. For instance, techniques like GB and G-Grad-CAM were found to generate visual explanations without proper training, raising concerns about their reliability. Therefore, while attribution map-based approaches in medical imaging are promising, they require careful evaluation and highlight the need for ongoing analysis to enhance the resilience, efficiency, replicability, and uniformity of these techniques in producing SM.

### 6.3. Insufficient Evaluation Metrics

Despite the progress made in applying XAI techniques to MI analysis, a notable gap remains in the evaluation of these methods. While none of the reviewed studies in this paper explicitly employed formal evaluation metrics for explainability, there do exist established evaluation methodologies in the broader XAI research landscape. For instance, Samek et al. [7,130] proposed a perturbation-based evaluation approach that measures the impact of removing or modifying input features on a model’s prediction to assess the relevance of explanations. Additionally, Nauta et al. [131] introduced a comprehensive framework for evaluating XAI methods, focusing on aspects like faithfulness, robustness, and complexity, thereby offering a structured methodology for comparing different XAI techniques.

This gap presents a valuable chance for prospective studies to advance the specialised evaluation metrics tailored to the unique needs of XAI. One major challenge in creating such metrics is the difficulty in creating a clear Baseline for clarification, given the inherently subjective nature of interpretability. As a result, this area of research holds significant potential, particularly as XAI in MI continues to evolve and the demand for more precise, contextually relevant evaluative criteria grows.

### 6.4. Complex Architecture

An Optimistic direction for ongoing studies is exploring the effectiveness of XAI methods within the high-level framework. Most current assessments of XAI techniques are based on simpler, shallower models, where methods Similar to the influence function can deliver reliable results. However, as models become deeper and more complex, these methods often struggle to deliver accurate explanations [181]. This raises critical Inquiries regarding the flexibility and dependability of existing XAI approaches when applied to advanced DL architectures. As such, there is a clear need to refine and enhance XAI methods to ensure they can maintain robust explainability as model complexity continues to increase.

### 6.5. Trade-Off Between Interpretability and Accuracy

In deep learning, a prevalent assumption is that there exists an intrinsic trade-off between interpretability and accuracy; in other words, highly accurate models are often less explainable, while more interpretable models tend to deliver lower accuracy. However, emerging studies dispute this perspective [182], proposing that enhancing interpretability could potentially improve accuracy. This revelation opens new avenues for future research aimed at developing explainable AI (XAI) methods that achieve a harmonious balance, combining superior explainability with outstanding performance.

### 6.6. Computational Cost

One of the major challenges in applying XAI techniques to medical imaging lies in the computational costs, which are particularly critical in real-time applications. Perturbation-based methods like LIME and SHAP, for example, are quite resource-intensive. These methods require multiple evaluations of the model and often involve retraining surrogate models, resulting in significant computational overhead [183]. This makes them less practical for scenarios where quick, time-sensitive decisions are needed, such as in emergency medical imaging. On the other hand, gradient-based approaches, including Grad-CAM, eigen Grad-CAM, G-Grad-CAM, XGrad-CAM, Grad-CAM++, Saliency Maps, Layerwise relevance Propagation, Randomised input sampling for explanation, Gradient Attention Rollout, Attention-based models, and DTD, are generally more productive. For example, Grad-CAM only requires one back step, which makes it suitable for applications needing real-time responses [59]. Likewise, techniques like Integrated Gradients (IG) and DTD effectively utilise gradient information to provide detailed explanations without compromising speed. Activation-based methods, like Class Activation Mapping, are also relatively optimised and efficient, as they rely on pre-calculated activation maps generated throughout the forward propagation. CAM involves computing a weighted sum of these maps, making it less demanding in terms of computation [57]. However, despite the efficiency of these methods, it’s essential to weigh the computational demands when selecting the most suitable XAI technique for MI tasks, particularly in settings where time is of the essence and computational resources are limited.

Recent work demonstrates that strategic optimizations can reduce perturbation-based explanation latency by 1–2 orders of magnitude without significant fidelity loss. For instance, FastSHAP achieves 100× speedup over KernelSHAP after a one-time training phase [184], while GPU-parallelized occlusion analysis processes 256 perturbations simultaneously critical for real-time deployment in emergency departments. Section 6.8 details clinically validated optimization strategies for time-critical settings.

### 6.7. Multimodal Data

A key area for future research in XAI lies in applying these techniques to multimodal datasets, especially within the realm of medical imaging. While most existing XAI approaches have primarily been tested on simpler, unimodal datasets, medical datasets often encompass more intricate patterns and diverse characteristics, presenting unique challenges for current methods. Multimodal data, such as X-rays, MRIs, CT scans, and microscopy images, require more advanced approaches that not only provide explanations but also effectively interpret these varied data sources. As a result, the XAI research community needs to create and evaluate innovative methods that can address the complexities inherent in multimodal medical datasets. This will ensure that XAI methods deliver robust, insightful, and meaningful explanations across different types of medical imagery, ultimately improving their applicability and reliability in real-world clinical settings.

### 6.8. Optimization Strategies for Real-Time XAI Deployment in Emergency Settings

While perturbation-based explainability methods such as LIME and SHAP provide reliable and theoretically grounded explanations, their computational cost remains a major limitation in emergency medical imaging scenarios, where rapid decision-making is essential [38,185]. In such time-critical settings, delays in generating explanations can hinder clinical usability and reduce the effectiveness of AI-assisted decision support systems. To address these challenges, several optimization strategies have been proposed in the literature to enable efficient and real-time XAI deployment.

**1. Algorithmic Approximations:** One key approach is to reduce the computational complexity of explanation methods through approximation techniques. Variants such as TreeSHAP enable efficient computation for tree-based models by leveraging model structure, significantly reducing computational overhead [38]. Similarly, learning-based explainers, such as FastSHAP, approximate Shapley values using surrogate neural networks, thereby enabling faster inference once trained [184]. In addition, adaptive sampling strategies for perturbation-based methods can dynamically reduce the number of required perturbations while maintaining explanation fidelity, improving efficiency in real-time applications.

**2. Hardware-Aware Acceleration:** Hardware-aware optimization techniques further enhance real-time performance. Parallel processing using GPUs or specialized accelerators allows simultaneous evaluation of multiple perturbations, significantly reducing execution time [186,187]. Additionally, model compression techniques such as quantization and pruning can reduce computational complexity and memory requirements without significantly compromising model accuracy, making them suitable for deployment in resource-constrained environments such as edge devices and emergency care units [188].

**3. Hybrid Explanation Pipelines:** To balance speed and interpretability, hybrid explanation strategies can be employed. In such approaches, fast gradient-based methods (e.g., Grad-CAM and its variants) are used for immediate, coarse-grained explanations, while more computationally intensive perturbation-based methods are applied selectively for detailed analysis or post-hoc verification [7]. This layered strategy enables efficient use of computational resources while maintaining explanation reliability.

**4. Clinical Workflow Integration:** Effective deployment of real-time XAI also requires alignment with clinical workflows. Strategies such as pre-computing explanations for common conditions, caching explanations for similar cases, and prioritizing computational resources based on clinical urgency can significantly improve system responsiveness [189]. Furthermore, integrating XAI systems into existing hospital infrastructure and decision-making pipelines ensures that explanations are delivered in a timely and clinically meaningful manner.

Overall, these optimization strategies contribute to reducing latency and improving the feasibility of deploying XAI systems in emergency medical settings. However, ensuring reliability, robustness, and clinical validation remains essential, particularly in high-risk scenarios where incorrect or delayed decisions may have critical consequences. All the details are given in Table 9.

## 7. Conclusions and Future Direction

In this comprehensive literature review, we explored the recent developments in XAI as applied to MI analysis. We identified and examined 18 different XAI techniques, providing detailed explanations of their definitions, core principles, and the mathematical frameworks they use in medical imaging contexts. This review also explores the challenges and limitations that each method faces, providing insights that can help researchers select the most suitable XAI approach based on their specific requirements. To ensure continued advancement in this field, it is crucial to prioritize the development of more robust evaluation metrics that accurately assess the effectiveness of XAI methods. Additionally, there is a pressing need to enhance the integration of XAI systems into clinical workflows, ensuring they align with real-world healthcare practices. Moreover, creating more sophisticated XAI architectures that maintain high performance while offering transparency is key to their broader adoption in clinical settings. Finally, exploring the combination of XAI with multimodal data and incorporating ensemble or hybrid AI models will not only increase the reliability of these systems but also enhance their practical application in clinical environments, ultimately making AI-driven medical imaging tools more accessible and trustworthy for healthcare professionals.

## Figures and Tables

**Table 2:** Gap analysis of existing XAI reviews versus our study.

Gap Area	Limitations in Prior Work	How This Manuscript Addresses the Gap
Evaluation metrics	Prior surveys rarely quantify or compare XAI evaluation approaches across clinical contexts	Synthesizes four evaluation dimensions (faithfulness, plausibility, robustness, clinical utility) with modality-specific protocol recommendations
Mathematical foundations	Many reviews lack technical depth in algorithmic mechanisms	Presents mathematical formulations for 18+ XAI methods with operational details in Section 2
Clinical translation	Few studies address workflow integration barriers	Discusses time-critical optimization strategies and clinician trust calibration in Section 6 and Section 7
Cross-modality applicability	Limited insight on method suitability across imaging types	Analyzes method performance across 8 modalities with disease-specific constraints (Section 4 and Section 5)

**Table 4:** Inclusion and exclusion criteria for selected publications.

Included Publications	Excluded Publications
Entire text accessible; Published between 2019 to 2024; Indexed in the referenced scientific databases; Research articles from conferences, journals, books, symposiums, and workshops across relevant domains; English-language studies focusing on XAI for medical care, including definition, interpretation, techniques, strategies, evaluation metrics, image analysis, image processing, and disease diagnosis.	Work-in-progress or unpublished studies; Non-English publications; Duplicate records; Studies analyzing XAI for image interpretation in non-medical imaging areas.

**Table 5:** Top publication venues for XAI research in medical imaging, summarizing the most recurring journals and conferences along with the number of papers published in each venue.

Rank	Publisher	Journal/Conference	No. of Papers
1	Elsevier	Computers in Biology and Medicine (CBM)	10
2	Elsevier	Computer Methods and Programs in Biomedicine (CMPB)	6
3	Elsevier	Biomedical Signal Processing and Control	5
4	Elsevier	Medical Image Analysis	10
5	Elsevier	Pattern Recognition	3
6	Elsevier	Other Elsevier Journals	8
7	IEEE	IEEE Transactions on Medical Imaging	10
8	IEEE	IEEE Journal of Biomedical and Health Informatics	8
9	Springer	MICCAI (International Conference on Medical Image Computing and Computer-Assisted Intervention)	5
10	Springer	Other Springer Journals	4
11	ACM	ACM Transactions on Multimedia Computing	5
12	ACM	ACM Conference Proceedings	3
13	Nature	Scientific Reports	5
14	Frontiers	Frontiers in Bioengineering and Biotechnology	3
15	PLOS	PLOS ONE/Medicine	3
16	CEUR-WS	CEUR Workshop Proceedings	10
17	Others	Diagnostics, IRBM, and additional venues	22

**Table 6:** Clinical decision guide: XAI evaluation protocol selection based on diagnostic context.

Clinical Scenario	Primary Evaluation Dimension	Recommended Metrics	Minimum Validation Requirement
Emergency triage (stroke, trauma)	Robustness + Clinical Utility	Deletion curve AUC, explanation latency	<2 s latency; ≥70% spatial overlap with expert annotations
Screening programs (mammography)	Plausibility + Faithfulness	Radiologist agreement (κ), insertion AUC	κ≥0.6 with 3+ radiologists; AUC > 0.75
Complex diagnostics (brain tumor subtyping)	All four dimensions	Full perturbation suite + multi-expert validation	κ≥0.75; robustness score > 0.85; prospective clinician study
Research/development phase	Faithfulness	Perturbation tests, sensitivity-norm	AUC > 0.70; sensitivity-norm < 0.3

**Table 9:** Clinical decision guide: XAI method selection based on time constraints and diagnostic requirements in emergency settings.

Clinical Scenario	Time Budget	Recommended XAI Method	Optimization Strategy	Validation Requirement
Code stroke (CT perfusion)	<1 s	Grad-CAM++	Pre-compute on GPU	Radiologist verification
			during scan acquisition	of penumbra localization
Trauma triage (X-ray)	1–3 s	TreeSHAP (if tree-based model)	Quantized model +	Comparison against
		or EigenGrad-CAM	batched perturbations	ATLS protocol decisions
Sepsis screening (multimodal)	3–10 s	Hybrid: Grad-CAM	Trigger Stage 2 only	ICU physician
		(Stage 1) + FastSHAP	if confidence <80%	acceptance study
		(Stage 2)		
Non-urgent follow-up	>10 s	Full LIME/SHAP	Standard implementation	INTRPRT
			implementation	compliance audit

## Data Availability

The raw data supporting the conclusions of this article will be made available by the authors on request.
